# Supplementary material for: Mauve/LYST limits fusion of lysosome-related organelles and promotes centrosomal recruitment of microtubule nucleating proteins
Source: Dev Cell. 2021 Apr 5;56(7):1000–1013.e6. doi: 10.1016/j.devcel.2021.02.019 (PMC8024676; doi:10.1016/j.devcel.2021.02.019)
Supplement: Document S1. Figures S1–S7 and Table S1 [file mmc1.pdf]

Developmental Cell, Volume *56*

Supplemental information

Mauve/LYST limits fusion of lysosome-related organelles and promotes centrosomal recruitment of microtubule nucleating proteins

Ramona Lattao, Hélène Rangone, Salud Llamazares, and David M. Glover

Figure S1

A

	mv^{ros}	mv^{3}	mv^{1}
mv^{ros}	Sterile		
mv^{3}	Semi-sterile	Lethal (second site)	
mv^{1}	Sterile	Sterile	Lethal (inversion)

B

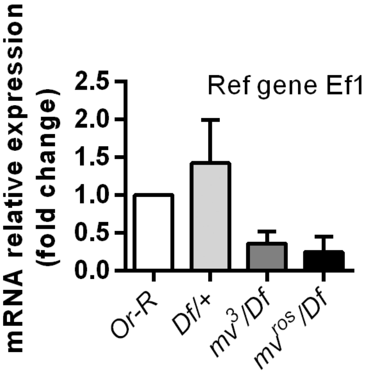

C **D**

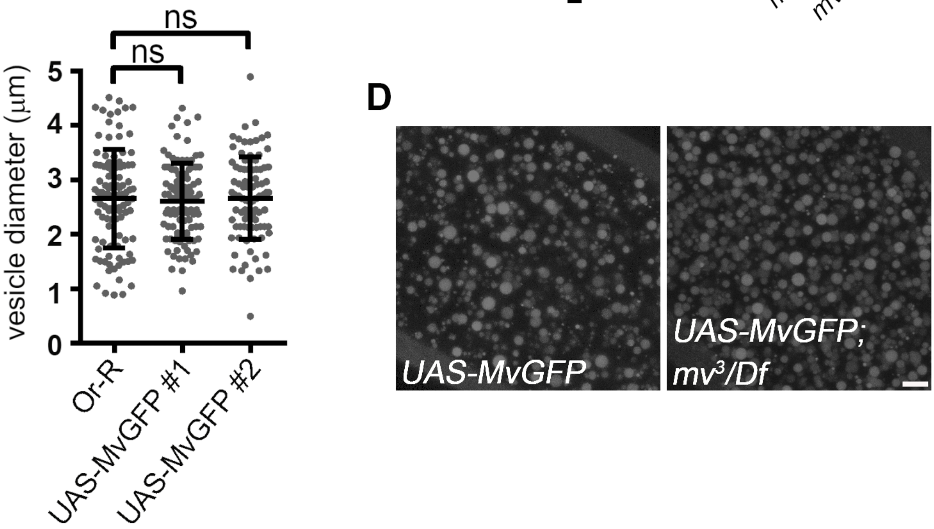

Figure S1 related to Figure 1. Identification of *mauve* alleles

(A) Phenotypes of different allelic combinations of *mauve* (*mv*)

(B) Quantitation of mauve mRNA level via qPCR in *Or-R*, *Df/+*, mv^3/*Df* and mv^{ros}/*Df*. The EF1 gene was used as reference.

(C) Vesicle diameter of YGs from *Or-R* embryos and 2 *UAS-MvGFP* lines (#1 and #2) driven by the maternal tubulin GAL4 promoter *P{matα4-GAL-VP16}* show no significative differences. Unpaired t test : *Or-R UAS-MvGFP #1* P value = 0,6425. *Or-R UAS-MvGFP #2* P value = 0,9430 (n=100, N=5)

(D) Examples of YGs from embryos of the indicated genotypes driven by *P{matα4-GAL-VP16}*. Scale bar =10 µm

Figure S2

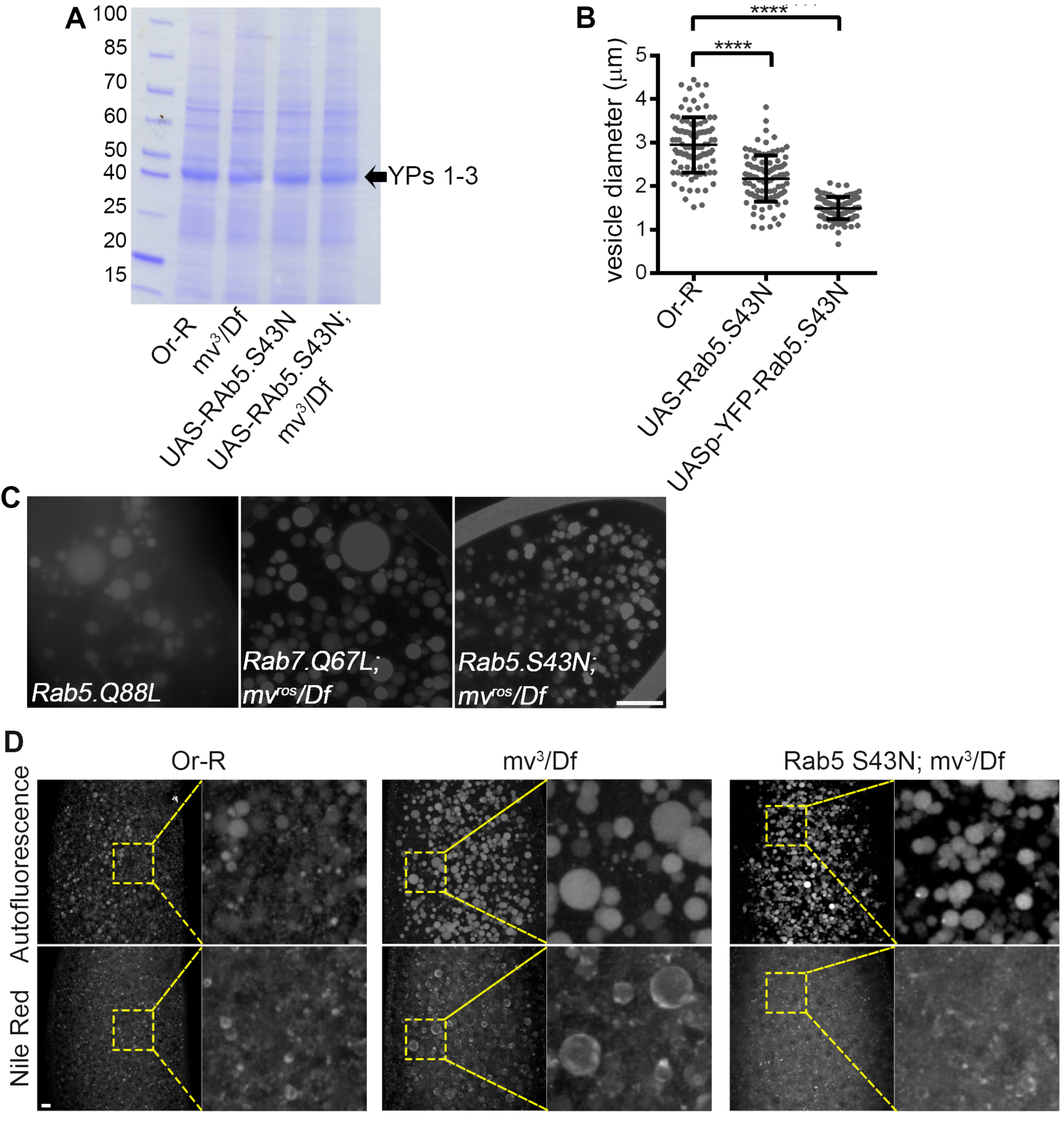
A
100
85
70
60
50
40
25
20
15
YPs 1-3
Or-R
mv3/Df
UAS-RAb5.S43N
UAS-RAb5.S43N; mv3/Df
B
vesicle diameter (µm)
0
1
2
3
4
5
****
****
Or-R
UAS-Rab5.S43N
UASp-YFP-Rab5.S43N
C
Rab5.Q88L
Rab7.Q67L; mvros/Df
Rab5.S43N; mvros/Df
D
Or-R
mv3/Df
Rab5 S43N; mv3/Df
Autofluorescence
Nile Red

Figure S2 related to Figure 2. Rab5.S43N does not affect yolk uptake and reduces fusion between YGs and LDs

(A) Coomassie staining of embryos of the indicated genotypes shows no defects in yolk uptake. 50µg of total embryonic extracts were loaded in each well. YPs band is the strongest band between 40 and 50 KDa. Expression of UAS transgenic constructs was driven by the maternal tubulin GAL4 promoter *P{matα4-GAL-VP16}*

(B) Vesicle diameter of YGs from embryos of the indicated genotypes to compare effect of 2 different Rab5.S43N transgenes with two different UAS promoters. Expression of UAS transgenic constructs was driven by *P{matα4-GAL-VP16}*. n=100, N=5 Unpaired t test: **** = P<0.0001. In all of our experiments, we used the UAS-Rab5.S43N transgene that has milder expression compare to the UASp driven transgene

(C) Examples of YGs from embryos of the indicated genotypes driven by *P{matα4-GAL-VP16}*. Scale bar =20 µm. See also Figure 2F, G

(D) Related to Figure 2H. Nile red staining of lipid droplets (LDs) and autofluorescent YGs in *Or-R*, *mv*^{3}*/Df* and *matα4-GAL-VP16/UAS-Rab5.S43N; mv*^{3}*/Df* embryos. In wild-type embryos, Nile Red show small puncta and sporadic localization around autofluorescent YG. In *mv*^{3}*/Df* there is a significative increase in Nile Red staining around YG that decreases in *matα4-GAL-VP16/UAS-Rab5.S43N; mv*^{3}*/Df* embryos. (n=50) Scale bar =10 µm

Figure S3

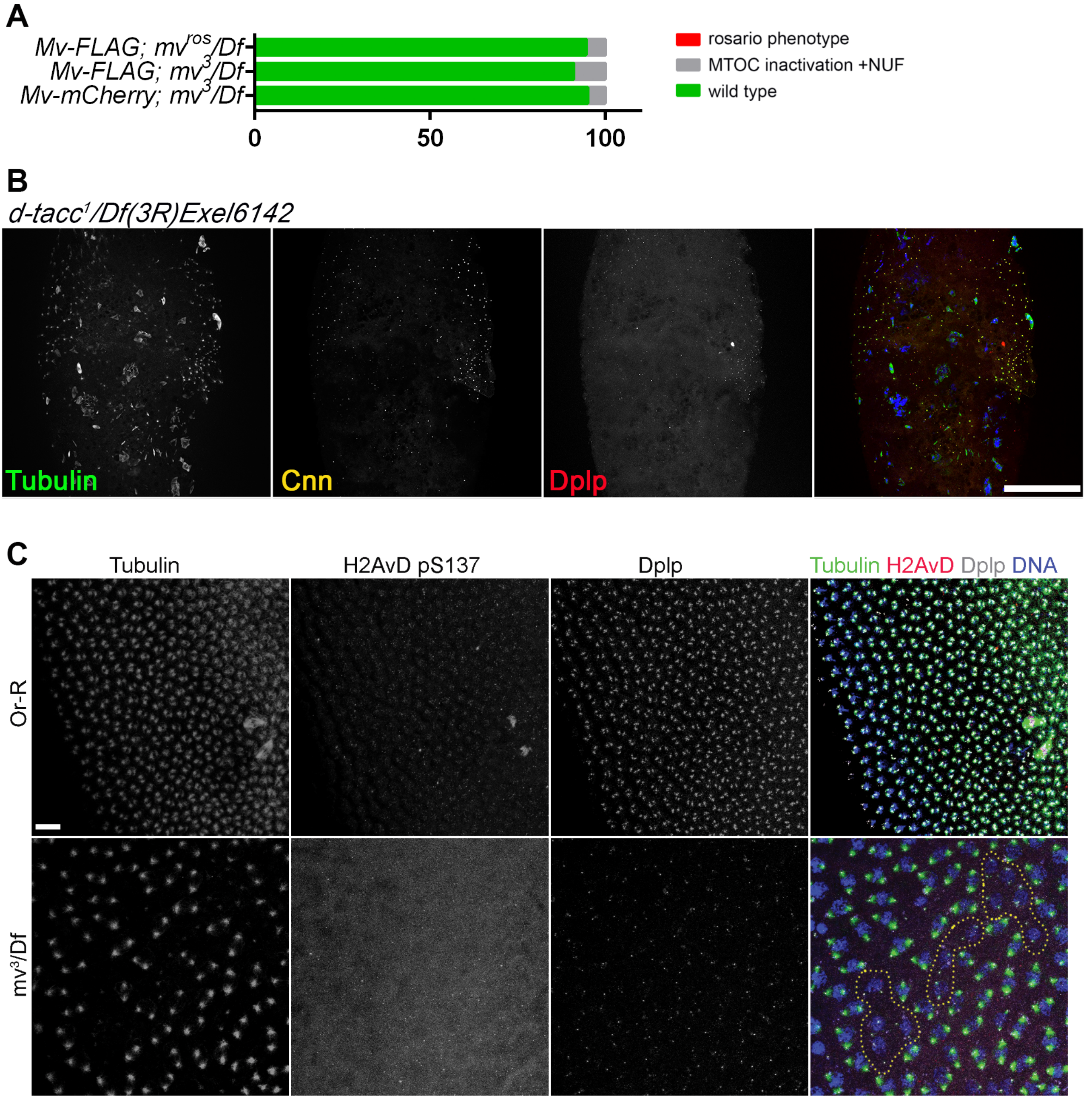
A
Mv-FLAG; mv^ros/Df
Mv-FLAG; mv^3/Df
Mv-mCherry; mv^3/Df
0
50
100
rosario phenotype
MTOC inactivation +NUF
wild type
B
d-tacc^1/Df(3R)Exel6142
Tubulin
Cnn
Dplp
C
Tubulin
H2AvD pS137
Dplp
Tubulin H2AvD Dplp DNA
Or-R
mv^3/Df

Figure S3 related to Figure 3. *d-tacc* mutants show a rosario-like phenotype and MTOC inactivation and NUF in mauve mutants are not consequences of DNA damage

(A) Frequencies (mean value) of different classes of phenotype after rescue with two different Mv transgenes (*Mv-mCherry* and *Mv-FLAG.*) n>100 for each genotype. See also Figure 3B

(B) Embryo from *d-tacc^1/Df(3R)Exel6142* female showing a rosario-like phenotype. Staining reveals α-tubulin, green; Centrosomin (Cnn), white; Dplp, red; and DNA, blue. Scale bar= 50µm

(C) *Or-R* and *mv^3/Df* embryos stained for α-tubulin (green), H2AvD pS137 (red), Dplp (grey) and DAPI (blue) show that MTOC inactivation in *mauve* mutants (yellow dotted area) is not a consequence of DNA damage. Scale bar = 10µm

Figure S4

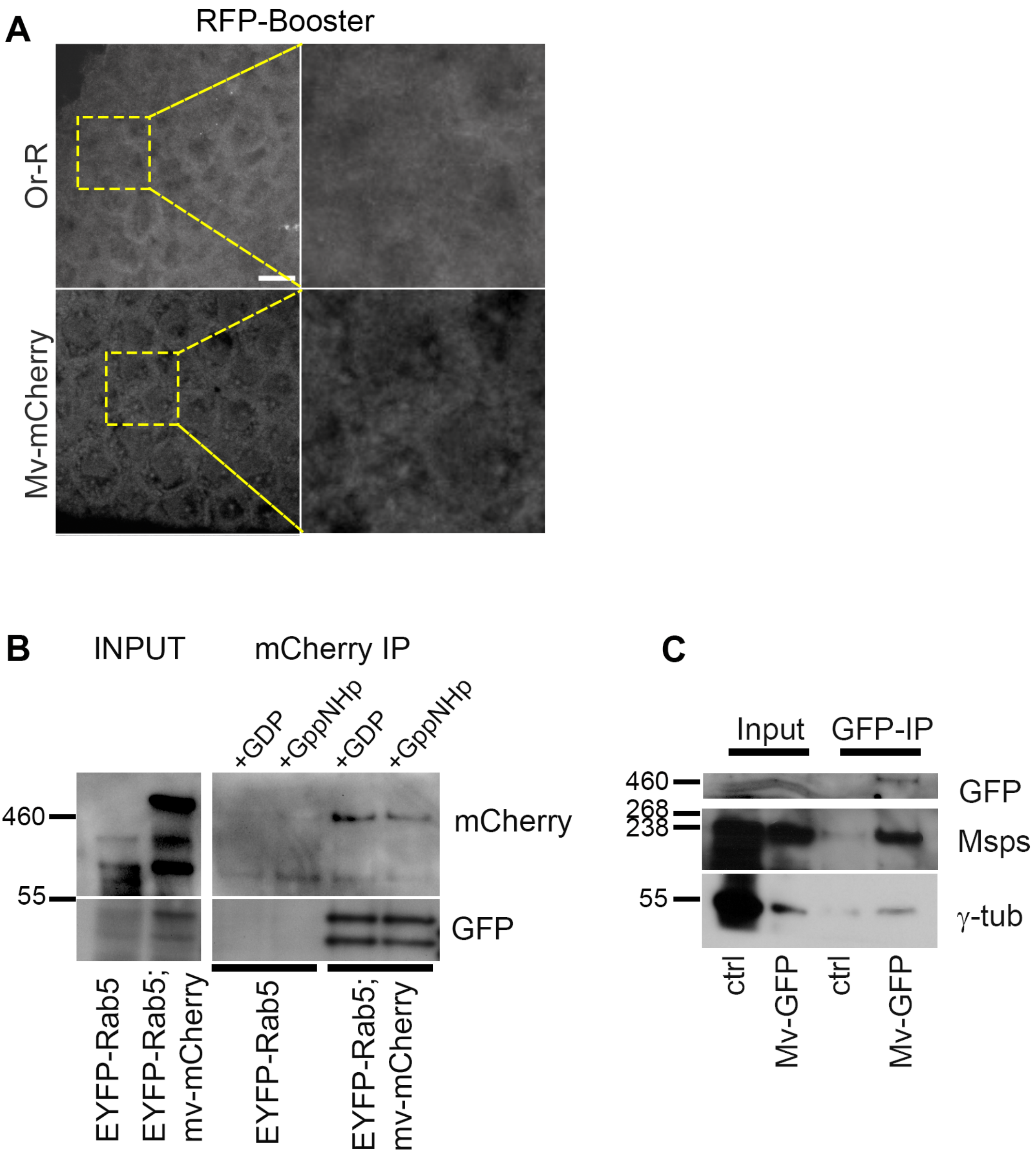
A
RFP-Booster
Or-R
Mv-mCherry
B
INPUT
mCherry IP
+GDP
+GppNHp
+GDP
+GppNHp
460
55
mCherry
GFP
EYFP-Rab5
EYFP-Rab5; mv-mCherry
EYFP-Rab5
EYFP-Rab5; mv-mCherry
C
Input
GFP-IP
460
268
238
55
GFP
Msps
γ-tub
ctrl
Mv-GFP
ctrl
Mv-GFP

Figure S4 related to Figure 4C, D. Mauve localizes at the mitotic spindle and interacts with Rab5, Msps and γ-tubulin

(A) Related to Figure 4C. Mv-mCherry localization in early embryonic divisions revealed with RFP-Booster Alexa Fluor® 568 (Chromotek). Mv-mCherry localizes all over the mitotic spindles and it enriches at spindle poles. Scale bar = 50µm

(B) Co-immunoprecipitation of Mv-mCherry complexes from 0-3h old embryos co-expressing Mv-Mcherry and EYFP- Rab5 (embryos expressing EYFP-Rab5 alone were used as control) in presence of GDP or the non-hydrolyzable GTP analog GppNHp. Importantly, EYFP-Rab5 is expressed under the endogenous promoter (*w[1118]; TI{TI}Rab5[EYFP]*, BDSC 62543) to avoid overexpression. Mv interacts *in vivo* with Rab5 and the interaction is not affected by the GDP/GTP conformation of Rab5.

(C) Co-immunoprecipitation of Mv-GFP complexes from DMEL cells in absence of detergent. wild-type DMEL cells were used as control. DMEL cells stable transfected with Mv-GFP under a metallothionein promoter were induced for 24h with 100µM $CuSO_4$ before Co-IP. Endogenous Msps and γ-tubulin were detected in the Mv-GFP IP but not in the control IP.

Figure S5

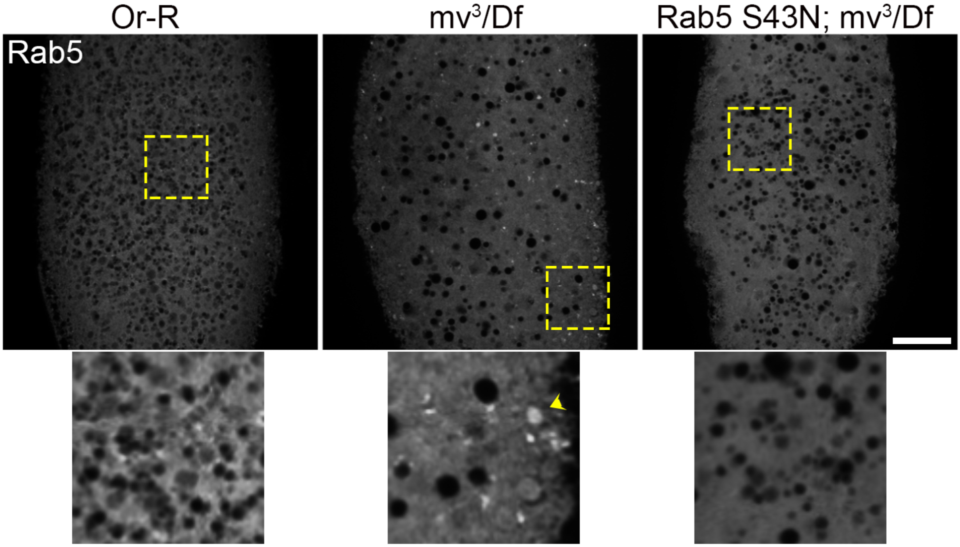

Figure S5 related to Figure 5 Rab5 accumulation around YGs in *Drosophila* embryos

In wild-type embryos, small puncta of Rab5 are interspersed within the YGs. In *mauve* mutant embryos, bigger Rab5 positive bodies accumulates in the vicinity of but are not incorporated into the YG “ghosts”. The accumulation of such bodies was suppressed by Rab5.S43N. Scale bar = 50 µm, insert = 3X

Figure S6

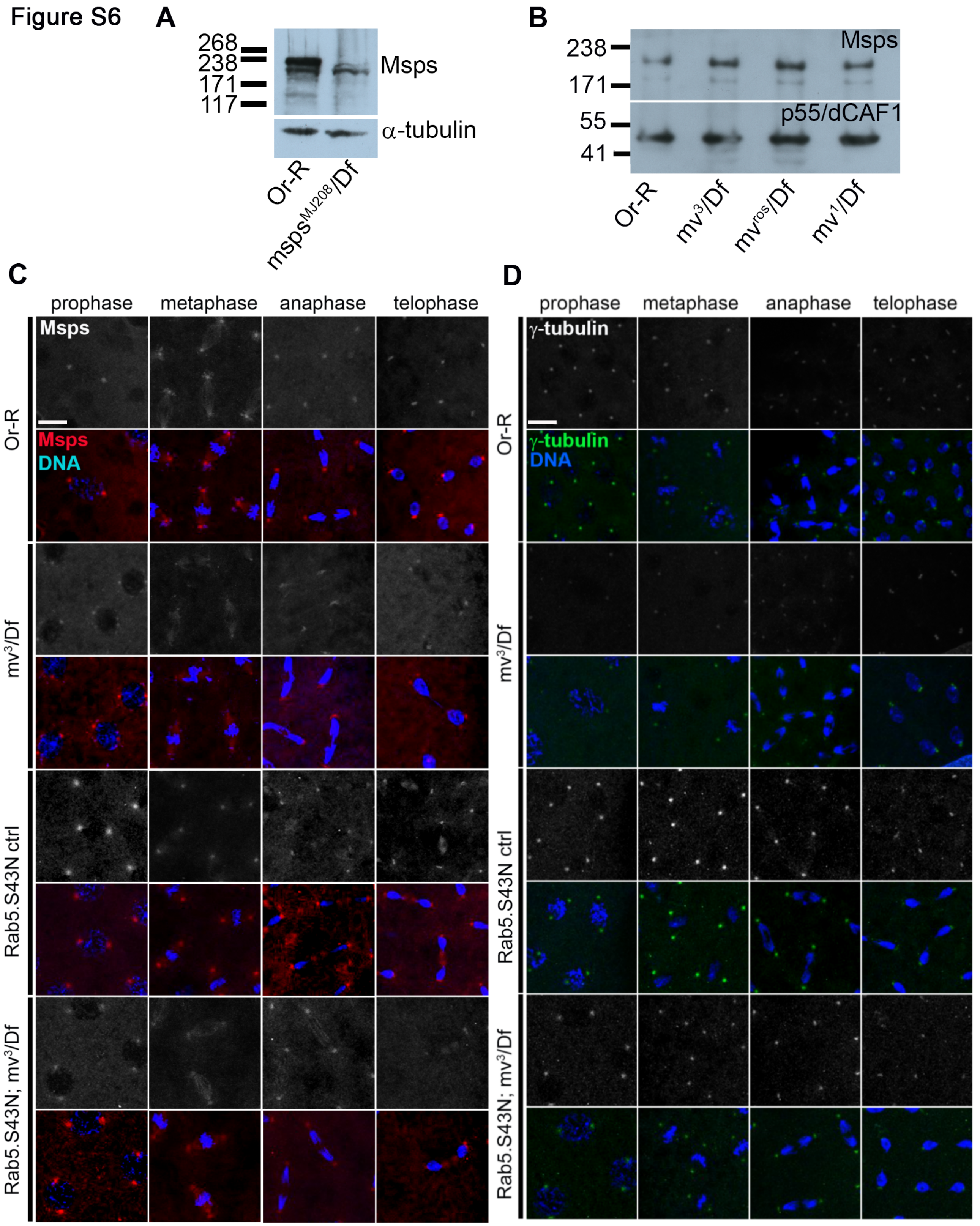
A
268
238
171
117
Msps
α-tubulin
Or-R
msps^MJ208/Df
B
238
171
55
41
Msps
p55/dCAF1
Or-R
mv^3/Df
mv^ros/Df
mv^1/Df
C
prophase
metaphase
anaphase
telophase
Msps
Msps
DNA
Or-R
mv^3/Df
Rab5.S43N ctrl
Rab5.S43N; mv^3/Df
D
prophase
metaphase
anaphase
telophase
γ-tubulin
γ-tubulin
DNA
Or-R
mv^3/Df
Rab5.S43N ctrl
Rab5.S43N; mv^3/Df

Figure S6 related to Figure 6. Characterization and staining with anti-Msps antibody

(A) Validation of Rabbit anti-Msps antibody raised against the 1350-1785 amino acid fragment of Msps. Western blots of ovary extracts from *Or-R* and $msps^{MJ208}/Df$ flies. $msps^{MJ208}$ is a hypomorphic allele of Msps. Note the significative reduction in the band corresponding to Msps. α-tubulin was used as loading control.

(B) Western blots of ovary extracts of *Or-R*, mv^{3}/Df, mv^{ros}/Df and mv^{1}/Df flies show that total levels of Msps are not affected in mauve mutants. p55/dCAF1 was used as loading control.

(C) Related to Figure 6C and showing all stages of mitosis stained to reveal Msps and DNA. Scale bar= 10μm

(D) Related to Figure 6D. Embryos from mothers of the indicated genotypes at representative stages of mitosis stained to reveal γ-tubulin and DNA. Scale bar= 10μm

Figure S7

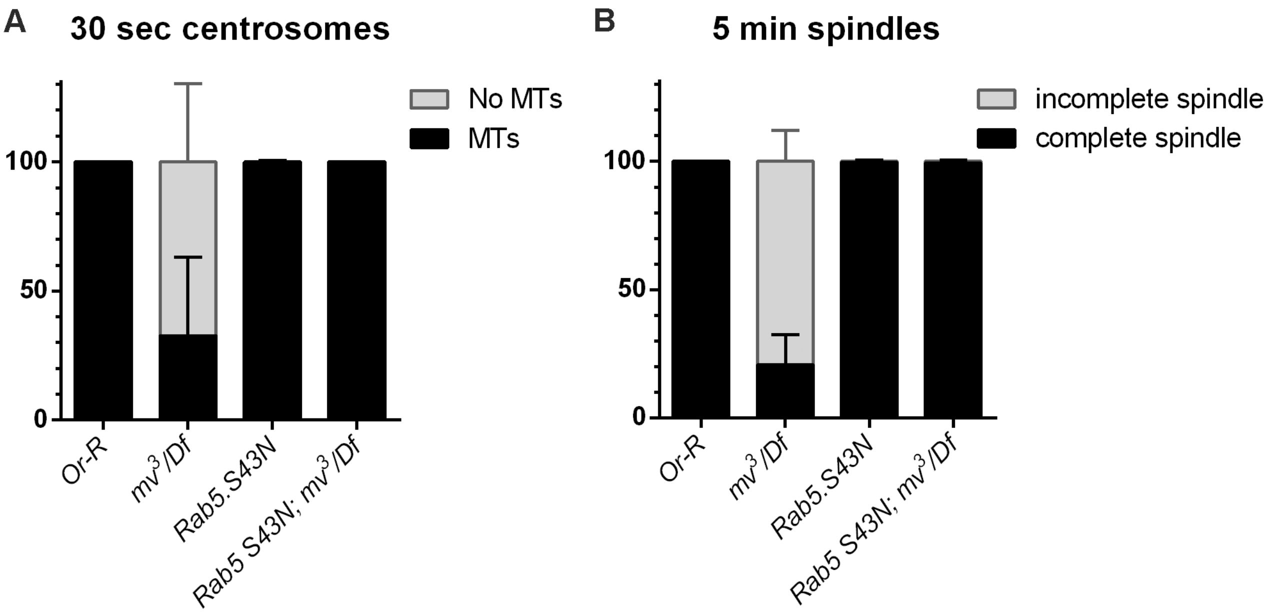
A
30 sec centrosomes
No MTs
MTs
100
50
0
Or-R
mv3/Df
Rab5.S43N
Rab5 S43N; mv3/Df
B
5 min spindles
incomplete spindle
complete spindle
100
50
0
Or-R
mv3/Df
Rab5.S43N
Rab5 S43N; mv3/Df

Figure S7 related to Figure 7. Quantification of MTs regrowth at 2 time points: 30 sec regrowth and 5 min regrowth

(A) Percentage of spindles with centrosome nucleating/non nucleating MTs after 30 sec regrowth. (*Or-R* n=124, N=4; *mv*^{3}*/Df* n=86, N=4; *Rab5.S43N* n=141, N=4; *Rab5.S43N mv*^{3}*/Df* n=184, N=5)

(B) Percentage of spindle with a full regrown spindle (complete spindle) or incomplete spindle after 5 min regrowth. (*Or-R* n=171, N=4; *mv*^{3}*/Df* n=160, N=4; *Rab5.S43N* n=126, N=3; *Rab5.S43N mv*^{3}*/Df* n=260, N=5)

Supplemental Table S1 related to STAR Methods. Primers used in this study

PRIMER NAME	SEQUENCE	SOURCE
2XFLAG_4XStop_KanaFF	GACTACAAGGACGATGCGACAAGGACTACAAGGACGATGACGACAAGTAATAATAGTGAGAGTCGTATTACATGGTCATAGC	Sigma-Aldrich
50ntffTC_1xFLAG	CAGAGGGGCTCTATGGAAATGCCCCGAAATTCCCTCAAATCGTCTACAAATGCTGCCCCGGCTGCTGCGACTACAAGGACGATGACGACAAG	Sigma-Aldrich
KanaTagRev	CTGTCATTGATATGCTTTGAGTTGAATTATTGAGTATTTTCCCATTAACATTAGTCCCGTCAAGTCAGCGTATT	Sigma-Aldrich
50CDSmCherryFF	CAGAGGGGCTCTATGGAAATGCCCCGAAATTCCCTCAAATCGTCTACAAAATGGTGAGCAAGGGCGAGG	Sigma-Aldrich
KanaStartRev_4Xstop_mCherryRev	GCTATGACCATGTAATACGACTCTCACTATTATTACTTGTACAGCTCGTCCATGCC	Sigma-Aldrich
mCherryEnd_4XStop_KanaFF	GCATGGACGAGCTGTACAAGTAATAATAGTGAGAGTCGTATTACATGGTCATAGC	Sigma-Aldrich
attBff	CGGCGGTGCGGGTGCCAGGGCGTGCCCTTGGGCTCCCCGGGCGCGTACTCCAC	Sigma-Aldrich
attBrev	GTGGAGTACGCGCCCGGGGGCCCAAGGGCACGCCCTGGCACCCGCACCGCGG	Sigma-Aldrich
qPCR_Mv_ff	GGATATCAAGCTCATTGC	Sigma-Aldrich
qPCR_Mv_rev	GTCCCAGAGCAAAGTTATG	Sigma-Aldrich
qPCR_EF1_ff	GCGTGGGTTTGTGATCAGTT	Sigma-Aldrich
qPCR_EF1_rev	GATCTTCTCCTTGCCCATCC	Sigma-Aldrich
Msps_1350-1785_Nter_ff	GGGGACAAGTTTGTACAAAAAAGCAGGCTTC TCG GCG GTG CGC GAG ATT GCT C	Sigma-Aldrich
Msps_1350-1785_Nter_rev	GGGGACCACTTTGTACAAGAAAGCTGGGTCcta CTC GAG AAT TAC GGC GTC ATA GTT C	Sigma-Aldrich
