# Supplementary material for: Mauve/LYST limits fusion of lysosome-related organelles and promotes centrosomal recruitment of microtubule nucleating proteins
Source: Dev Cell. 2021 Apr 5;56(7):1000–1013.e6. doi: 10.1016/j.devcel.2021.02.019 (PMC8024676; doi:10.1016/j.devcel.2021.02.019)
Supplement: Document S2. Article plus Supplemental information [file mmc8.pdf]

Developmental Cell

Article

Mauve/LYST limits fusion of lysosome-related organelles and promotes centrosomal recruitment of microtubule nucleating proteins

Graphical abstract

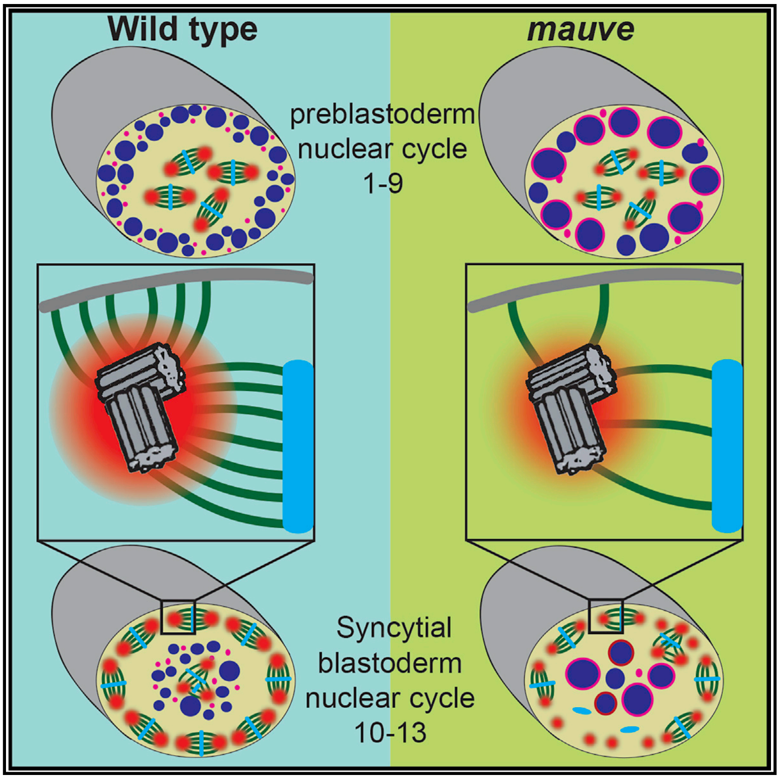

Highlights

- *Drosophila* Mauve regulates lysosome-related organelle and lipid droplet fusion
- Mauve regulates PCM recruitment and microtubule nucleation from centrosomes
- Mauve interacts with Rab5, Msps, and γ-tubulin
- Mauve/LYST opposes Rab5, which promotes vesicle fusion affecting PCM recruitment

Authors

Ramona Lattao, Hélène Rangone, Salud Llamazares, David M. Glover

Correspondence

rl489@cam.ac.uk (R.L.),
dmglover@caltech.edu (D.M.G.)

In brief

Lattao et al. investigate the role of Mauve, the *Drosophila* homolog of the gene LYST, which is mutated in the poorly understood Chediak-Higashi syndrome. They show that Mauve/LYST regulates vesicle fusion and microtubule nucleation at centrosomes, revealing an important connection among vesicles, centrosome homeostasis, and microtubule nucleation.

Lattao et al., 2021, Developmental Cell *56*, 1000–1013
April 5, 2021 © 2021 The Author(s). Published by Elsevier Inc.
https://doi.org/10.1016/j.devcel.2021.02.019

CellPress
OPEN ACCESS

Developmental Cell

Article

Mauve/LYST limits fusion of lysosome-related organelles and promotes centrosomal recruitment of microtubule nucleating proteins

Ramona Lattao,[1,4,*] Hélène Rangone,[1] Salud Llamazares,[2] and David M. Glover[1,3,*]
[1]University of Cambridge, Department of Genetics, Downing Street, Cambridge CB23EH, UK
[2]Institute for Research in Biomedicine (IRB Barcelona), The Barcelona Institute of Science and Technology, Parc Cientific de Barcelona, C/ Baldiri Reixac 10, 08028 Barcelona, Spain
[3]Division of Biology and Biological Engineering, California Institute of Technology, 1200 E, California Blvd, Pasadena, CA 91125, USA
[4]Lead contact
*Correspondence: rl489@cam.ac.uk (R.L.), dmglover@caltech.edu (D.M.G.)
https://doi.org/10.1016/j.devcel.2021.02.019

SUMMARY

Lysosome-related organelles (LROs) are endosomal compartments carrying tissue-specific proteins, which become enlarged in Chediak-Higashi syndrome (CHS) due to mutations in *LYST*. Here, we show that *Drosophila* Mauve, a counterpart of *LYST*, suppresses vesicle fusion events with lipid droplets (LDs) during the formation of yolk granules (YGs), the LROs of the syncytial embryo, and opposes Rab5, which promotes fusion. Mauve localizes on YGs and at spindle poles, and it co-immunoprecipitates with the LDs' component and microtubule-associated protein Minispindles/Ch-TOG. Minispindles levels are increased at the enlarged YGs and diminished around centrosomes in *mauve*-derived mutant embryos. This leads to decreased microtubule nucleation from centrosomes, a defect that can be rescued by dominant-negative Rab5. Together, this reveals an unanticipated link between endosomal vesicles and centrosomes. These findings establish Mauve/LYST's role in regulating LRO formation and centrosome behavior, a role that could account for the enlarged LROs and centrosome positioning defects at the immune synapse of CHS patients.

INTRODUCTION

Autosomal recessive Chediak-Higashi syndrome (CHS) results from a mutation in the lysosomal trafficking regulator (LYST) or CHS1 gene and leads to partial albinism, neurological abnormalities, and recurrent bacterial infections (Kaplan et al., 2008; Ward et al., 2000). CHS cells have giant lysosome-related organelles (LROs), compartments that, in addition to lysosomal proteins, contain cell-type-specific proteins (Marks et al., 2013). LROs include melanosomes, lytic granules, MHC class II compartments, platelet-dense granules, basophil granules, azurophil granules, and pigment granules of *Drosophila*. Whether the giant LROs of CHS form through the excessive fusion of LROs (Kypri et al., 2007; Oliver and Essner, 1975; Willingham et al., 1981) or by inhibition of their fission (Durchfort et al., 2012; Perou et al., 1997) is unclear.

The compromised immune system in CHS is associated with enlarged LROs in natural-killer (NK) cells. NK cells normally become polarized with centrosomes close to their contact site with antigen-presenting cells, the immunological synapse (IS). Despite the formation of a mature IS in CHS NK cells, centrosomes do not correctly polarize and the enlarged LROs neither converge at the centrosome nor translocate to the synapse (Chiang et al., 2017; Gil-Krzewska et al., 2016, 2018). Such findings could reflect defective microtubule (MT) organization by the centrosomes in CHS cells, and while some groups describe CHS centrosomes to nucleate fewer MTs (Boxer et al., 1979; Oliver and Zurier, 1976), others report normal MT numbers, lengths, and distributions (Frankel et al., 1978; Ostlund et al., 1980; Pryzwansky et al., 1985). Thus, the consequence of mutation in LYST for centrosome and MT function is unclear.

Drosophila's LYST counterpart is encoded by *mauve* (*mv*) (CG42863) (Figure 1A). *mv* mutants show a characteristic eye color due to larger pigment granules, defective cellular immunity through large phagosomes, and enlarged starvation-induced autophagosomes, indicating several types of LRO are affected (Rahman et al., 2012). The embryo's LROs are the yolk granules (YGs), which provide nutrition and energy during early development (Fagotto, 1995). YGs are produced and stored in the egg chamber when the yolk proteins (YPs) of follicle cells are internalized by clathrin-mediated endocytosis and trafficked through the endocytic pathway of the growing oocyte (Brennan et al., 1982; DiMario and Mahowald, 1987; Liu et al., 2015). YGs are present at the periphery of the egg until the early nuclear division cycles of the syncytial embryo, when they translocate to the interior as nuclei migrate to the embryo's cortex in nuclear division cycles 8 and 9 (Foe and Alberts, 1983). Nurse cells of the egg chamber also supply eggs with endoplasmic-reticulum-derived lipid droplets (LDs), which store maternally provided proteins and neutral lipids

 © 2021 The Author(s). Published by Elsevier Inc.
This is an open access article under the CC BY license (http://creativecommons.org/licenses/by/4.0/).

CellPress
OPEN ACCESS

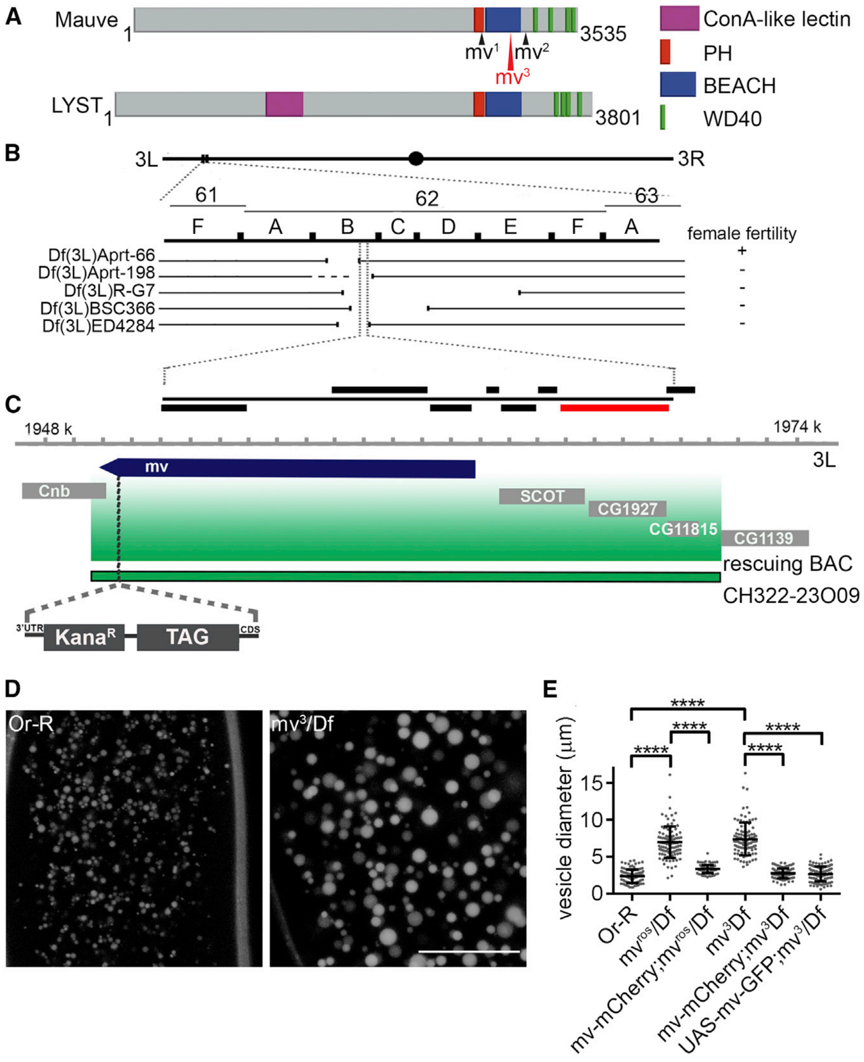

Figure 1. Mutations in *mv* result in enlarged YGs

(A) Comparison of human LYST and *Drosophila* Mauve protein domains. Positions of known *mv* mutations are shown against the *Drosophila* protein (mv^3, red, is newly identified here). Downstream of a pleckstrin homology (PH) domain of approximately 100 residues lies a “beige and CHS” (BEACH) domain of about 300 residues and a series of WD40 repeats.

(B) Deficiency mapping of 61F-63A indicating female fertility observed when deficiencies were heterozygous with mv^{ros} and mv^3 alleles. The expanded interval shows all genes with *mv* in red.

(C) Genomic region (green shading) encompassing *mauve* carried in the BAC CH322-23O09 used for genomic rescue. BAC recombineering introduced an in-frame mCherry or FLAG tag at the C-terminus of the coding sequence (CDS) followed by 4 stop codons and a kanamycin resistance gene ($kana^R$) upstream of the *mv* 3′UTR.

(D) Examples of autofluorescent LROs (YGs) in *Or-R* and mv^3/Df embryos. Scale bar, 50μm.

(E) Diameters of LROs (YGs) in *Or-R*, *mv/Df*-derived embryos, and *mv/Df*-derived embryos with rescue transgenes, *Mv-mCherry* or *UAS-Mv-GFP / P{matα4-GAL-VP16}*: *Or-R*, 2.38 ± 0.09 μm; mv^{ros}/Df, 6.96 ± 0.21 μm; mv^3/Df, 7.39 ± 0.22 μm; *Mv-mCherry*; mv^{ros}/Df, 3.32 ± 0.05 μm; *Mv-mCherry*; mv^3/Df, 2.74 ± 0.07 μm; *UAS-Mv-GFP* mv^3/Df, 2.68 ± 0.1 μm). n = 100, mean±SEM. Unpaired t test: ****$p < 0.0001$

for energy and membrane biosynthesis (Cermelli et al., 2006; Kühnlein, 2012).

Here, we reveal Mauve’s role in regulating LRO/YGs and MT nucleation from centrosomes through the maternal effect lethal (MEL) phenotypes of two new mutant alleles of *mauve*, $mv^{rosario}$ (mv^{ros}) and mv^3. We show that embryos derived from mutant *mv* females have enlarged YGs that fuse with LDs, and this can be reverted by reducing Rab5 activity. *mv*-derived embryos also show compromised MT nucleation leading to defects in the embryo’s mitotic cycles and cytoskeletal organization. Moreover, a requirement for Mauve in regulating MTs through the TACC/Msps pathway suggests a role for endosomal trafficking in the recruitment or maintenance of pericentriolar material (PCM) components at centrosomes.

RESULTS

Maternal effect mutants of *Drosophila mv* display enlarged YGs in the syncytial embryo

LYST/CHS1 protein is characterized by pleckstrin homology (PH), BEACH, and WD40 motifs in its C-terminal part, which are conserved in its *Drosophila* counterpart, Mauve (Figure 1A). The syncytial embryo’s YGs are prominent, maternally provided LROs required for embryonic development suggesting there should be MEL alleles of *mv*, i.e., *mv* mutant mothers whose embryos would not develop. We identified two such mutations: *fs(3)ros (rosario)*, which we previously mapped within a cytological interval encompassing *mv* (Kasravi et al., 1999) and found to be allelic with a second site mutation in *l(3)dre6* (Sliter et al., 1989). We localized these mutations to a smaller cytogenetic interval and showed by complementation tests that they are mv^1 alleles (STAR methods; Figures 1B and S1A).

mv produces a 14-Kb primary transcript, comprising 18 exons and encoding a 3,535 amino-acid protein. The mv^3 sequence revealed a G to A mutation in the splice acceptor site of intron 15–16. We confirmed that intron 15–16 was retained in mv^3 mutants by amplifying this region from mv^3/Df transcripts and sequencing (data not shown). We were unable to sequence mv^{ros} due to complex DNA rearrangements. However, semiquantitative qRT-PCR showed *mv* mRNA levels were significantly reduced in both mv^{ros}/Df and mv^3/Df compared with those in *Df* /+ larvae (Figure S1B).

In line with the enlarged LRO size seen in various organisms and cell types upon mutation in LYST and its homologs, we observed enlarged autofluorescent YGs in 100% of *mv*-derived embryos (Figure 1D). Whereas wild-type YGs measured

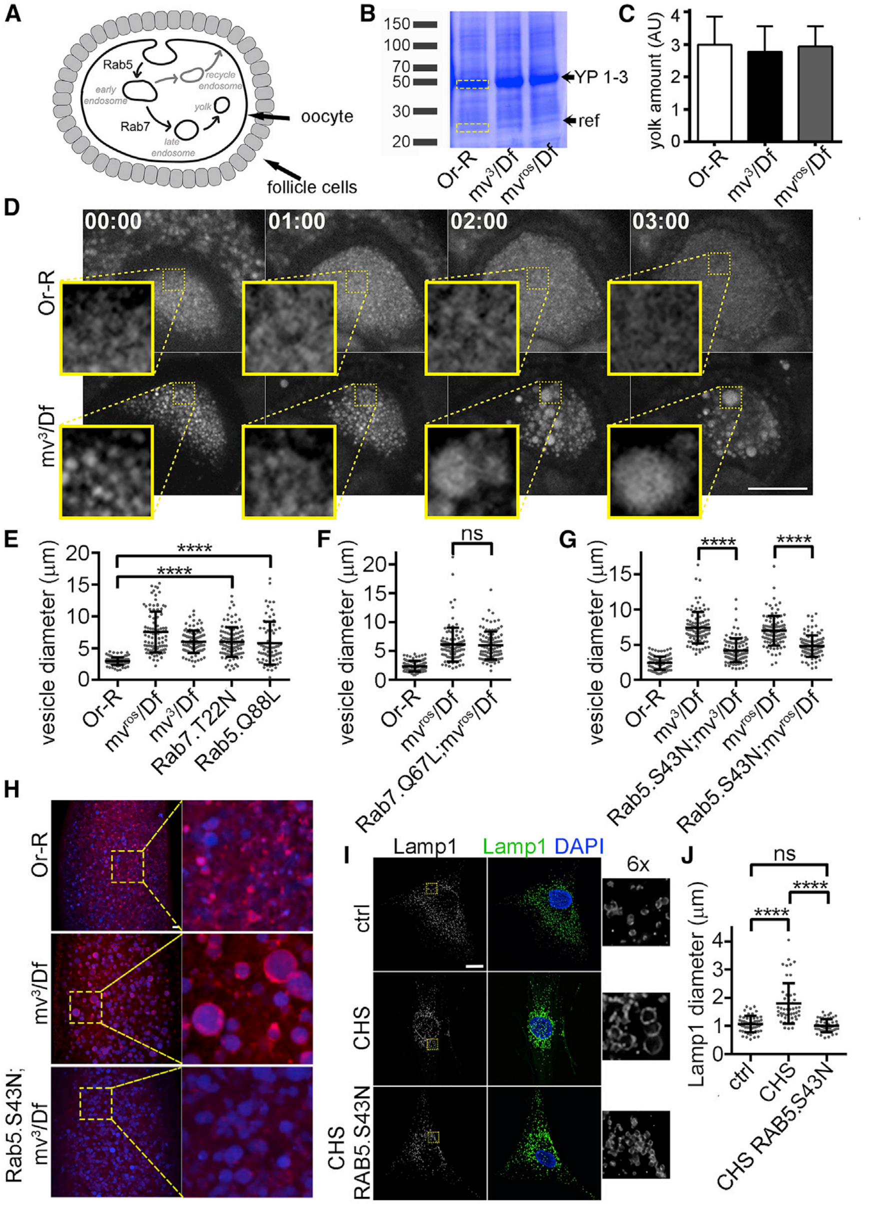

Figure 2. LRO defects in *mv* oocytes, embryos and CHS fibroblasts

(A) Schematic of YG formation in oocytes initiated by endocytosis of components secreted by the follicular cells, showing involvement of Rab5 and Rab7.

(B) Coomassie stain of ovarian extracts showing YP biosynthesis is not affected in *mv* mutants.

(C) Quantitation of YP Coomassie-stained band intensity from (B) (arbitrary units [AU]; mean ± SD of 3 replicates). AU represents intensity ratio between YP band (yellow upper box) and reference band below YP band (ref, yellow lower box) in 2B.

(D) Time frames from time-lapse videos (hh:mm; see also Videos S1 and S2) of autofluorescent YG formation in *Or-R* and *mv^3/Df* oocytes. Scale bar, 50 μm; inset, 4× enlargement.

(E) Enlargement of YGs in *mv* mutants and in dominant-negative Rab7 (Rab7.T22N, 5.97 ± 0.23 μm), and constitutively active Rab5 (Rab5.Q88L, 5.80 ± 0.39 μm) embryos. Constructs expression driven by *P{matα4-GAL-VP16}*. n = 100, mean ± SEM. Unpaired t test: ****p < 0.0001. See also Figure S2C.

(F) Constitutively active Rab7.Q67L does not reduce YG sizes in *mv*-derived embryos (*+/UASp-YFP-Rab7.Q67L;mv^{ros}/Df*, 6.12 μm ± 0.3, n=100; *matα4-GAL-VP16/UASp-YFP-Rab7.Q67L;mv^{ros}/Df*, 5.98 ± 0.24 μm, n = 100, mean ± SEM). Unpaired t test, p = 0,7154. See also Figure S2C.

(G) Dominant-negative Rab5.S43N reduces YG size in *mv*-derived embryos (*matα4-GAL-VP16/UAS-Rab5.S43N*; *mv^3/Df*, 4.19 ± 0.17 μm; *matα4-GAL-VP16/UAS-Rab5.S43N*; *mv^{ros}/Df*, 4.78 ± 0.16 μm, n = 100, mean±SEM). Unpaired t test: ****p < 0.0001. See also Figure S2C.

(H) Nile red staining of LDs (red) and autofluorescent YGs (blue) in *Or-R*, *mv^3/Df*- and *matα4-GAL-VP16/UAS-Rab5.S43N*; *mv^3/Df*-derived embryos. (n = 50) Scale bar, 10 μm. See also Figure S2D.

(I) Human fibroblasts from a control individual, from a CHS patient, and from CHS fibroblasts constitutively expressing EGFP-RAB5A.S43N, all stained to reveal lysosomal marker, Lamp1, (green) and DNA (blue). Scale bar = 10 μm.

(J) Lamp1 vesicle diameter is reduced by dominant-negative RAB5 (control, 1.07 ± 0.04 μm n = 51; CHS, 1.8 ± 0.1 μm n = 51; CHS RAB5.S43N, 1.01 ± 0.04 μm. n = 50, mean ± SEM). Unpaired t test: ****p < 0.0001

2.38 ± 0.09 μm in diameter, *mv* mutant YGs were up to 3 times larger (6.96 ± 0.21 μm in *mv^{ros}/Df*- and 7.39 ± 0.22 μm in *mv^3/Df* -derived embryos; Figure 1E). To confirm that these enlarged YGs resulted from lack of Mauve, we introduced Mauve transgenes expressed from either the endogenous promoter (*Mv-mCherry*) or a maternal germline driver (*UAS-Mv-GFP*) and found they restored YGs to wild-type size (*Mv-mCherry*; *mv^{ros}/Df*, 3.32 ± 0.05 μm; *Mv-mCherry*; *mv^3/Df*, 2.74 ± 0.07 μm; *UAS-Mv-GFP*; *mv^3/Df*, 2.68 ± 0.1 μm; Figures 1C, 1E, and S1D).

Conserved regulation of LRO size by Mauve/LYST

YP (1–3) comprise the major protein traffic of oocyte endocytosis to become the principal content of YGs (Figure 2A) (Bownes and Hames, 1977; Tsuruhara et al., 1990; Warren and Mahowald, 1979). We found no significant differences in amounts of YPs 1–3 between wild-type and *mv* ovaries (Figures 2B and 2C) or embryos (Figure S2A) indicating that Mauve is required neither for yolk biosynthesis nor uptake but only influences YG size.

When we followed YG formation during vitellogenesis by time-lapse microscopy of stage 9 oocytes (Figure 2D; Videos S1 and S2), we observed that whereas autofluorescent yolk-containing vesicles did not develop beyond a specific size in wild-type oocytes (Video S1; Figure 2D), excessive vesicle fusion led them to become markedly larger in *mv^3/Df* oocytes (Video S2; Figure 2D).

As YP endocytosis requires Rab5 in early, and Rab7 in late endosomes (Liu et al., 2015) (Figure 2A), we asked if Mauve might

CellPress
OPEN ACCESS

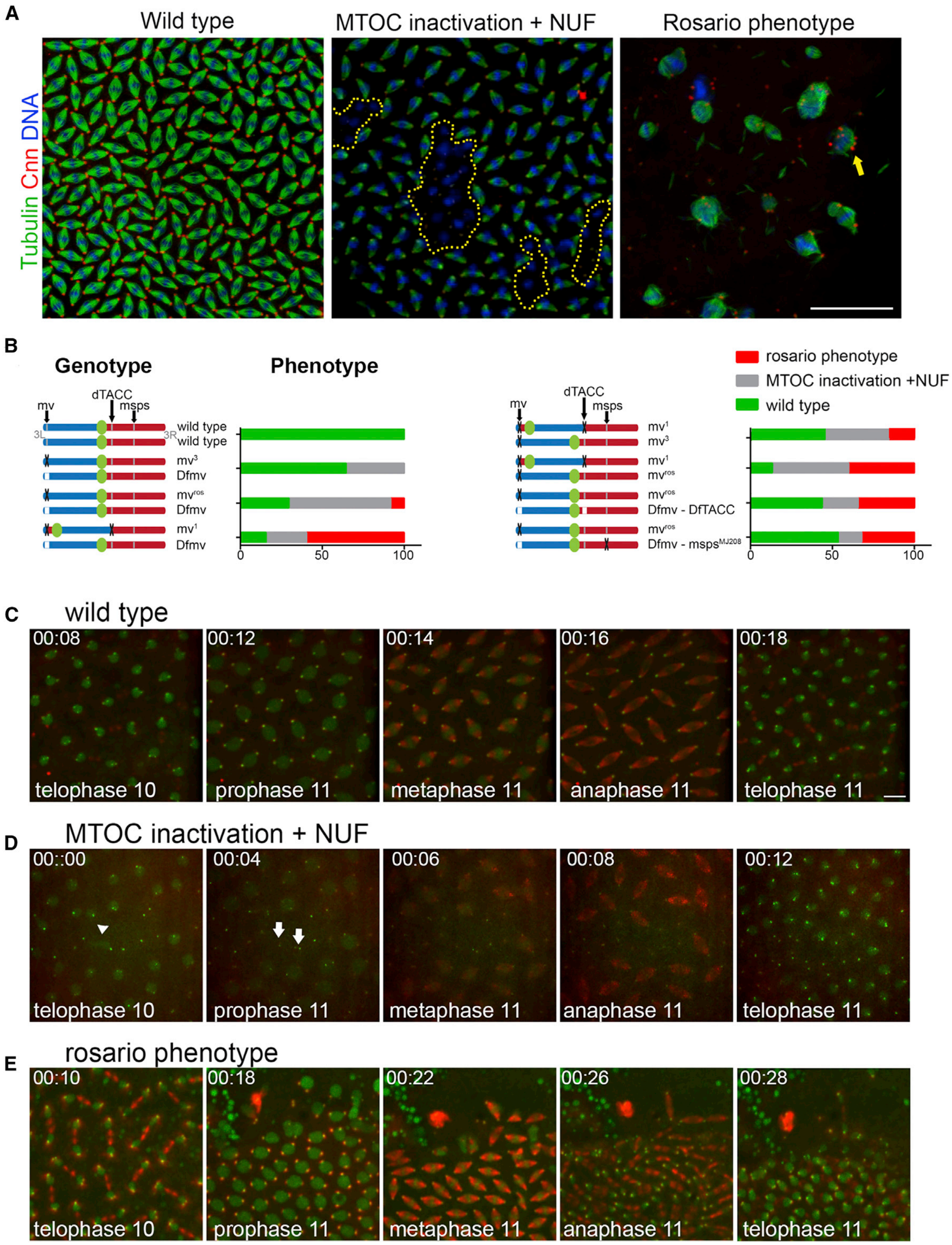

Figure 3. *mv* mutants exhibit maternal effect MT defects and genetically interact with *d-tacc* and *msps*

(A) Fixed preparations of wild-type and *mv*-derived embryos stained to reveal α-tubulin, green; Centrosomin (Cnn), red; and DNA, blue. Embryos with mitotic spindles at the cortex scored in 3 groups: wild-type showing synchronous, equally spaced spindles; MTOC inactivation and NUF (yellow dotted areas); and rosario with multiple free centrosomes (yellow arrow). Although sporadic NUF is observed in wild-type embryos, here NUF was scored when it involved more than 3 juxtaposed nuclei. Scale bar, 50 μm.

(legend continued on next page)

interact with the endocytic pathway to regulate vesicle size. To this end, we expressed constitutively active Rab5 (Rab5.Q88L) (Liu et al., 2015; Stenmark et al., 1994) or inactive Rab7 (Rab7.T22N) (Girard et al., 2014; Liu et al., 2015; Vanlandingham and Ceresa, 2009) and in both cases observed enlargement of YGs to a similar extent as in *mv* mutants (Rab5.Q88L = 5.8 ± 0.39 μm; Rab7.T22N = 5.97 ± 0.23 μm) (Figures 2E and S2C). We then asked whether dominant-negative Rab5 (Rab5.S43N) or constitutively active Rab7 (Rab7.Q67L) could restore *mv* YGs toward their wild-type dimensions. Whereas Rab7.Q67L had no effect upon the size of *mv* YGs (5.98±0.24 μm in *matα4-GAL-VP16*; *UASp-YFP-Rab7.Q67L*; *mv^{ros}/Df*) (Figures 2F and S2C), Rab5.S43N led to a significant reduction in their diameter (4.19±0.17 μm in *matα4-GAL-VP16*; *UASp-Rab5.S43N*; *mv^{3}/Df* and 4.78 ± 0.16 μm in *matα4-GAL-VP16*; *UASp-Rab5.S43N*; *mv^{ros}/Df*) (Figures 2G, S2B, and S2C) without affecting YP uptake in *mv* mutant backgrounds (Figure S2A). Thus, these findings suggest that enlargement of LROs is promoted by Rab5 through vesicle fusion whereas Mauve opposes Rab5 to limit vesicle fusion events.

In addition to YGs, *Drosophila* embryos also have a maternal dowry of LDs (approximately 0.5-μm diameter), which store lipids and proteins, including histones and cytoskeleton proteins, that are essential for embryo development (Cermelli et al., 2006; Kühnlein, 2012). In wild-type embryos, LDs (revealed by Nile Red dye) are small punctate bodies, distinct from the autofluorescent YGs. However, *mv*-derived mutant embryos had greatly reduced numbers of these puncta and, instead, showed a substantial increase in Nile Red staining around YGs. Strikingly, this could also be rescued by expressing Rab5.S43N (Figures 2H and S2D). Thus, LDs appear to be incorporated into the enlarged YGs of *mv*-derived embryos in a Rab5-dependent manner.

To determine whether Mauve's role was conserved, we investigated the consequences of expressing dominant-negative human RAB5 (RAB5.S43N) in fibroblasts derived from a CHS patient (GM02075, Coriell Institute) bearing a single base duplication resulting in a truncated form of LYST (Barbosa et al., 1996). We stably transformed such CHS fibroblasts with EGFP-RAB5A.S43N (here termed *CHS1RAB5.S43N*) and compared their lysosomes with those of the *CHS1* parental cells and wild-type fibroblasts revealed by the lysosomal marker, Lamp1. In accord with our observations on *Drosophila mv* mutant YGs, we found that *CHS1* fibroblasts had enlarged lysosomes (1.8 ± 0.1-μm diameter) compared with those in wild-type cells (1.07 ± 0.04 μm) and that expression of dominant-negative RAB5A.S43N restored Lamp1-containing vesicles of *CHS* cells (1.01 ± 0.04 μm) toward wild-type diameter (Figures 2I and 2J). Thus, we conclude that the regulatory interaction between Mauve/LYST and the endocytic pathway is conserved between *Drosophila* and human cells.

mv mutants exhibit maternal effect MT defects and genetically interact with *d-tacc* and *msps*

A small fraction (8.3% ± 6.02%, mean±SEM) of *mv^{ros}/Df*-derived embryos displayed aberrant mitotic spindles of different sizes associated with varying amounts of DNA, abnormal chromosome segregation, and multiple centrosomes often in chains, leading us to name the mutant *rosario*—rosary beads (Figures 3A and 3B). Closer examination of *mv^{ros}/Df*-derived embryos revealed that the greater proportion (62.1% ± 11.2%) reached the syncytial blastoderm stage but exhibited localized MT organizing center (MTOC) inactivation and loss of contact between nuclei and centrosomes at the cortex leading nuclei to sink into the interior of the embryo—nuclear fallout (NUF) (Figures 3A and 3B). We did not observe the rosario phenotype in *mv^{3}/Df* -derived embryos, of which 35.4% ± 5.2% showed MTOC inactivation and NUF (Figure 3B). These mitotic phenotypes could be rescued by *Mv-mCherry* or *Mv-FLAG* transgenes in the mutant background (Figure S3A). Thus, a mutation in *mv* leads to unexpected defects in the behavior of the mitotic apparatus in the nuclear division cycles of the syncytial embryo.

To understand these mitotic defects, we undertook time-lapse imaging of cortical nuclear division cycles using RFP-tagged Tubulin to follow MTs and GFP-tagged Fzr to follow centrosomes and nuclei. In wild-type embryos, nuclei undergo synchronous migration to the embryo's cortex in cycles 9–10 and divide in synchrony until cellularization at cycle 14 (Figure 3C; Video S3). In *mv*-derived embryos, the nuclear division cycles were synchronous until syncytial blastoderm, when small clusters of nuclei lost their division synchrony concomitantly with diminished MT nucleation by centrosomes leading to NUF (Figure 3D; Video S4). In rosario embryos, we initially observed cortical migration of nuclei followed by an abrupt flow of cytoplasm from within the embryo toward its surface displacing cortical nuclei toward the embryo's poles (Figure 3E; Video S5). This cytoplasmic flow led to the surfacing of yolk nuclei, which normally get left behind in the embryo's interior, fall out of the mitotic cycle but continue to endo-reduplicate while their associated centrosomes continue to divide (Riparbelli and Callaini, 2003). Those yolk nuclei ectopically positioned at the cortex of rosario embryos continued to undergo their characteristic nuclear and centrosomal cycles (Video S5).

The rosario phenotype resembles aspects of the MEL phenotype of *d-tacc* (CG9765), which encodes the *Drosophila* counterpart of the MT-associated, transforming acidic coiled-coil protein 3 (TACC-3; Gergely et al., 2000). *d-tacc*-derived embryos show a failure of pronuclear fusion to initiate the nuclear division cycles or, should this succeed, failure of cortical migration of nuclei and free centrosomes. Those rare *d-tacc^{1}* mutant embryos reaching cycle 10 display a similar movement of yolk nuclei to the cortex associated with multiple free centrosomes as in rosario embryos (Figure S3B). The possibility that *d-tacc* might interact genetically

(B) Schematics of chromosome 3 showing different alleles *of mv* in combination with indicated mutant alleles and deficiencies. The frequencies (mean value) of the different classes of the phenotype are shown alongside the diagrams of the genotypes. Quantification is carried out in fixed embryos. N > 100 for each category. *mv^{1}* /+ has a complete wild-type phenotype. See also Figure S3A.

(C, D, and E) Time frames from time-lapse videos of embryos with *Ub-Tubulin-RFP* and *Ub-Fzr-GFP* transgenes to illustrate the three phenotypic classes (see also Videos S3, S4, and S5). Scale bar, 10 μm. Arrows in D show the beginning of MTOC inactivation. Figure 5E shows the first cycle in the initiation of development of the rosario phenotype. Several rounds of centrosome duplication are required for chains of centrosomes to develop and can be tracked in Video S5. Genotypes: C = *Ub-tubulin-RFP Ub-Fzr-GFP*; +/+ D= *Ub-tubulin-RFP Ub-Fzr-GFP*; *mv^{3}/Df* E = D = *Ub-tubulin-RFP Ub-Fzr-GFP*; *mv^{ros}/Df*.

with *mv* was suggested by the strong rosario phenotype of mv^1, which results from an inversion (*In(3LR)264*) with one breakpoint in *mv* and the other in *d-tacc* (Figure 3B) (Rahman et al., 2012). When we placed *In*(3LR)*264* mv^1 against the chromosome carrying mv^3, the phenotype of mv^3 was significantly enhanced such that 15.5 ± 7.2% (mean ± SEM) of embryos showed the rosario phenotype. The proportion of rosario embryos also increased substantially (35.22% ± 4.8%) when *In*(3LR)*264* mv^1 was placed against mv^{ros}. Moreover, when we placed *In*(3LR) *264* mv^1 against a deficiency that completely deletes *mv*, the frequency of embryos with the rosario phenotype increased to almost 60% (59.57% ± 8.79%). To confirm this genetic interaction between *mv* and *d-tacc*, we generated a recombinant chromosome carrying a *mv* deficiency (*Df(3L)R-G7* - referred to as *Dfmv*) and a deficiency uncovering *d-tacc* (*Df(3R)Exel6142* - referred to as *Df-d-tacc*) and found that when placed against mv^{ros}, this enhanced the proportion of rosario embryos to 30.45% ± 2.95% (Figure 3B). As the D-TACC protein occurs in a complex with the MT-associated protein Minispindles (Msps, CG5000) (Lee et al., 2001), we also generated a recombinant chromosome carrying the *mv* deficiency and the $msps^{MJ208}$ mutant allele. This recombinant also enhanced the phenotype of mv^{ros} such that 32.7% ± 0.6% of mv^{ros}/*Dfmv*-$msps^{MJ208}$-derived embryos displayed the rosario phenotype (Figure 3B). Together, these genetic observations indicate that when MT dynamics are compromised by mutations affecting the D-TACC: Msps complex, the loss of Mauve results in the rosario phenotype, suggesting that Mauve contributes to regulating MT dynamics and thereby the spatial organization of the syncytium.

Mauve localizes around LROs in *Drosophila* oocytes and at centrosomes and mitotic spindles in the syncytial embryo

Next, we asked whether Mauve's dual role, implied by the above findings, would be reflected in its localization. To date, the localization of LYST counterparts has been elusive, with the exception of *Dictyostelium discoideum* LvsB, which is present on late and post-lysosomes (Kypri et al., 2007). When we examined the ovaries, eggs, and early embryos of Mv-mCherry transgenic flies, we found Mauve in the follicular epithelium as an aggregate at the apical side of cells facing the developing oocyte (Figure 4A) and in punctate bodies close to the oocyte membrane, the site of YP endocytosis (data not shown). In the early stages of vitellogenesis, Mv-mCherry aggregates were too small to accurately localize but, at later stages, YGs had Mv-mCherry enveloping the autofluorescent yolk, suggesting an association with membranous structures (Figure 4B). This accords with Mauve's predicted membrane localization that would result from its phospholipid-binding PH and BEACH domains. In addition, we observed that in syncytial embryos, Mv-mCherry was associated with the mitotic spindle; colocalized with the centrosomal marker DPlp (arrows in Figure 4C); and was present in small punctate bodies clouding around the spindle poles (arrowheads in Figures 4C and S4A) consistent with a mitotic function of Mauve.

Mauve associates with subsets of vesicle trafficking and spindle-associated proteins

The genetic interactions of *mv* with *d-tacc* and *msps* and with dominant-negative Rab5 led us to ask whether any of these proteins are associated with Mauve in wild-type syncytial embryos. To this end, we immunoprecipitated Mv-mCherry from 3-h collections of *Drosophila* embryos expressing this transgene for characterization by mass spectrometry (Figure 4D; Tables S2). Notably, this revealed three classes of proteins: motors among which, the most prominent were Dynein/Dynactin and their associated subunits; proteins of endocytosis, including Clathrin and its regulatory subunits, Rab5 and Rab7; and proteins associated with MT minus ends at centrosomes, including γ-tubulin ring complex components, Asp and Msps but not D-TACC. We confirmed the interaction of Mauve with Rab5 by co-immunoprecipitating Mv-mCherry from 0–3 h old embryos co-expressing Mv-mCherry and Rab5-EYFP in presence of GDP or the nonhydrolyzable GTP analog GppNHp. Both proteins were present in the immunoprecipitate irrespective of the GTP-bound or GDP-bound conformation of Rab5 (Figure S4B). We also confirmed Mauve's interactions with endogenous Msps and γ-tubulin, which co-immunoprecipitated in Mv-GFP complexes from cultured *Drosophila melanogaster* (DMEL) cells (Figure S4C). Collectively, these results indicate that endocytic vesicle trafficking proteins co-purify with Mauve as do PCM components and other proteins associated with MT minus ends, pointing toward physical connections between Mauve, vesicle trafficking proteins, and centrosomal regulators of MT nucleation.

The genetic and physical interactions of Mauve and Rab5 prompted us to investigate Rab5 localization in *mv* mutants (Figure 5A). In wild-type embryos, small Rab5-positive structures were dispersed around spindles whereas in *mv* mutant embryos, there were larger Rab5-positive aggregates that became significantly reduced after Rab5.S43N expression (Figure 5A). Similarly, we found an accumulation of Rab5-positive bodies in the vicinity of, but not incorporated into, the YG "ghosts" in fixed preparations of *mv*-derived embryos. The accumulation of such bodies was suppressed by Rab5.S43N (Figure S5). In confirmation of these findings, we observed a similar pattern of RAB5 endosomes in human fibroblasts. In contrast to the small RAB5-positive endosomes of control fibroblasts, CHS fibroblasts had more intensely staining RAB5-positive vesicles and this phenotype could be rescued by overexpression of human RAB5A.S43N (Figure 5B). Thus, loss of Mauve/LYST function appears to result in the fusion of LROs accompanied by an accumulation of Rab5-specific bodies in a Rab5-dependent manner.

Mauve is required for the centrosomal association of Msps

To further investigate the relationship between Mauve and centrosomal proteins, we examined the behavior of Msps in the nuclear division cycles of syncytial embryos derived from *mv* mutant females. We raised an anti-Msps antibody (Figure S6A) that revealed total Msps levels not to be affected in *mv* mutant ovaries (Figure S6B). In wild-type embryos, Msps associates with centrosomes in prophase, peaking at metaphase, when it also associates spindle MTs. This spindle association diminishes at anaphase when centrosomal staining also becomes weaker in line with previous reports (Cullen et al., 1999; Figures 6A, 6C, and S6C). In mv^3/*Df*-derived embryos, spindle MT-associated Msps was comparable with that in wild-type embryos at metaphase but centrosomal Msps was considerably weaker (Figures 6A–6C and S6C). To quantify the centrosome versus spindle

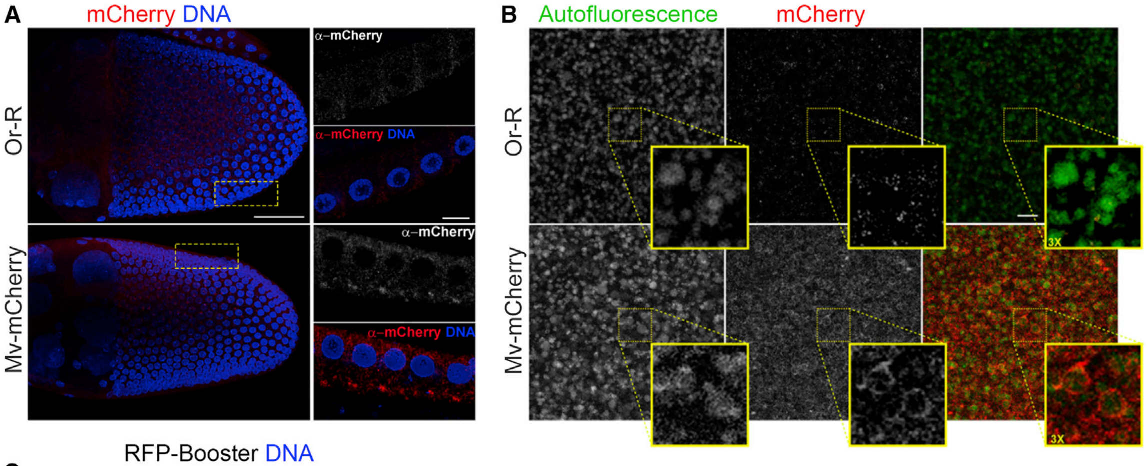

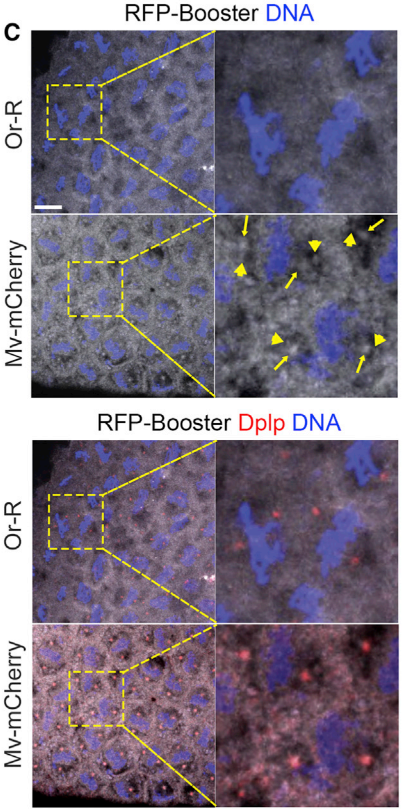

D

	Pull down #1		Pull down #2	
**GENE**	**Score**	**Peptides**	**Score**	**Peptides**
**MAUVE (CG42863)**	**17163**	**285**	**14899**	**307**
**PCM COMPONENTS**				
Mini spindles (CG5000)	1436	28	466	11
Gamma Tubulina at 37C (CG17566) [a]	1172	17	630	15
Polo (CG12306)	1155	27	425	16
Abnormal spindles (CG6875)	465	11	841	29
Gamma Tubulin ring protein 91 (CG10988)	447	6	281	10
Gamma Tubulin ring protein 84 (CG3917)	303	5	378	10
Gamma Tubulin ring protein 75 (CG6176)	225	6	137	5
Spindle defective 2 (CG17286)	120	1	53	1
Gamma Tubulin at 23C (CG3157)	113	2	100	3
Gamma Tubulin ring protein 163 (CG5688)	106	1	150	3
Gamma Tubulin ring protein 128 (CG9201)	80	2	82	3
**MOTOR SUBUNITS**				
Dynein heavy chain 64C (CG7507)	7303	129	3157	80
Kinesin-like protein at 3A (CG8590)	767	12	350	8
Dynein light intermediate chain (CG1938)	686	11	77	3
Dynactin1, p150 subunit (CG9206)	457	7	466	13
Sperm-specific dynein intermediate chain 1 (CG9580)	228	3	66	3
Non-claret disjunctional (CG7831)	200	3	123	4
Cytoplasmic dynein light chain 2 (CG5450)	221	3	-	-
**VESICLE TRAFFICKING**				
Clathrin Heavy Chain (CG9012)	1790	37	768	20
Arl1 (CG6025)	157	2	72	1
Shibire/Dynamin (CG18102)	880	20	961	24
Comatose/NSF1 (CG1618)	329	5	291	9
Otefin (CG5581)	788	9	207	4
Sar1 (CG7073)	727	12	139	4
Rod (CG1569)	1704	32	13l73	32
Atlasin (CG6668)	274	6	109	4
Ap1-μ (CG9388)	162	3	88	3
Ap1-γ (CG9113)	154	2	38	1
Zw10 (CG9900)	528	8	736	18
Rab5 (CG3664)	286	6	78	1
Rab7 (CG5915)	133	3	73	1

a=present in control pull down with 1 peptide and score 40

Figure 4. Mauve protein localizes around LROs in *Drosophila* oocytes and at mitotic spindles in early embryonic divisions

(A) Localization of Mauve in stage 10 egg chambers stained to reveal Mv-mCherry, red; DNA, blue. Mv-mCherry has a polarized localization in follicular epithelial cells, enriched on the side facing the developing oocyte where yolk components are secreted before uptake into the oocyte. Scale bar, 50 μm; zoom, 10 μm.

(B) Mauve localization in freshly dissected, unfixed mature eggs. YGs are autofluorescent in the green, but not, the red channel. Mv-mCherry localizes around the YGs. Scale bar, 10 μm.

(C) Mv-mCherry localization in early embryonic divisions revealed by RFP-Booster Alexa Fluor 568 (Chromotek, gray), Dplp (red), and DAPI (blue). Mv-mCherry localizes all over the mitotic spindles and is enriched at the poles where it colocalizes with DPlp. Such localization was observed at all stages of mitotic division (data not shown). *Or-R* flies were used as controls (n = 50). Scale bar, 10 μm. See also Figure S4A.

(D) Mass spectrometric identification of proteins co-immunoprecipitating with Mv-mCherry from 0–3 h embryos (full datasets in Table S2).

difference in Msps association, we measured the ratio of the fluorescence intensity at single centrosomes and their associated half spindles. This confirmed the diminution of centrosomal Msps in mv^3/*Df*-derived embryos in comparison with that in wild-type embryos (mv^3/*Df*, 1.18 ± 0.07; wild-type, 2.76 ± 0.17) (Figure 6B).

These findings led us to consider that other vesicle trafficking molecules might participate in regulating the centrosomal association of Msps. We considered a potential role of Rab5 because Rab5.S43N could rescue the enlarged YG phenotype (Figures 2G, 2H, and S2C); Rab5 co-purified with Mauve (Figures 4D and S4B); and our laboratory had previously described a Rab5

CellPress
OPEN ACCESS

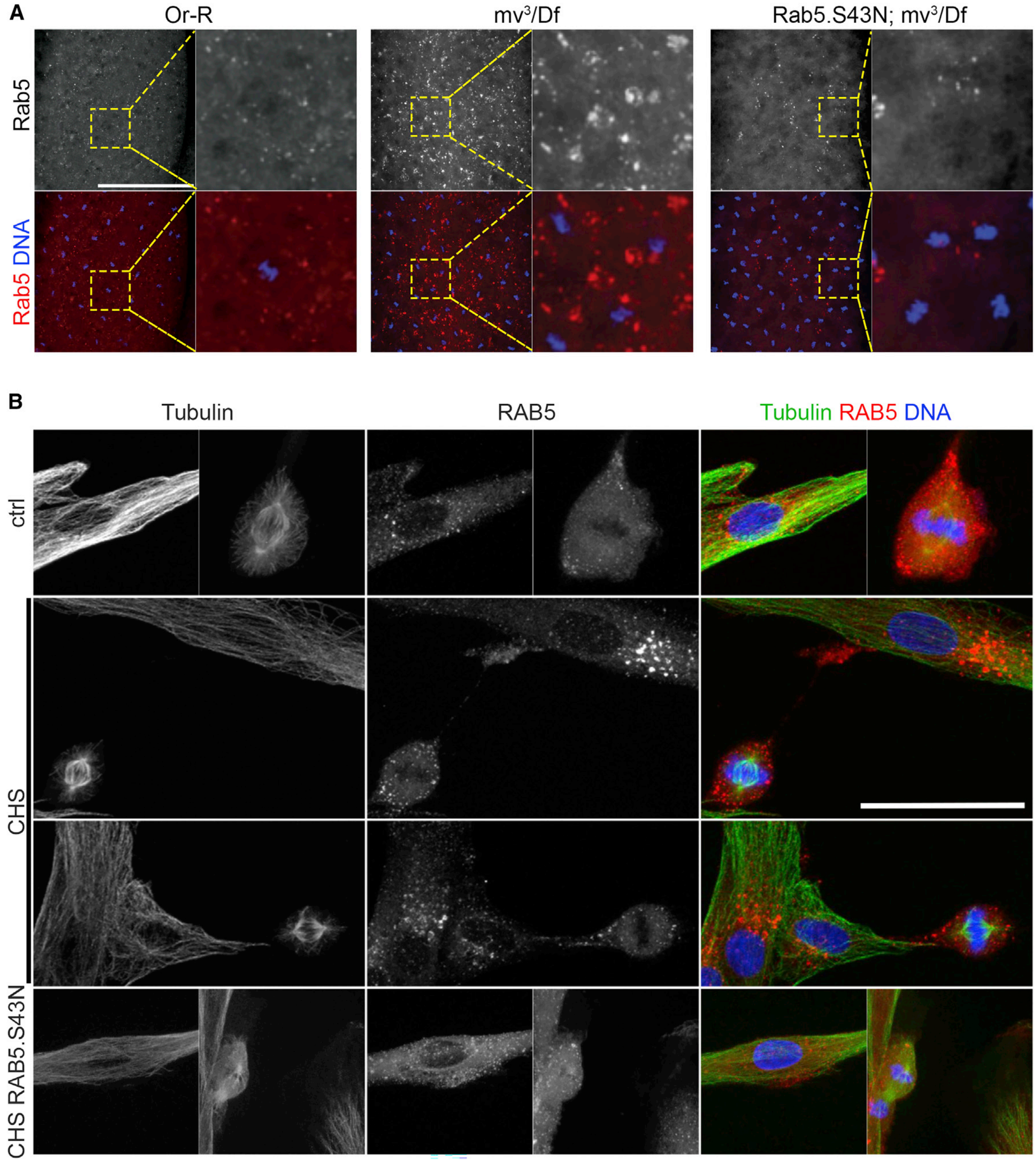

Figure 5. Rab5-positive endosomes in *mv*-derived *Drosophila* embryos and CHS human fibroblasts

(A) Localization of Rab5 (red) and DNA (blue) in *Or-R*, *mv*^{3}*/Df and matα4-GAL-VP16/UAS-Rab5.S43N*; *mv*^{3}*/Df*-derived embryos. In wild-type embryos, small Rab5 positive structures are dispersed in the syncytium, and in *mv*^{3}*/Df*-derived mutant embryos, aggregates of Rab5-positive structures are present and these structures are significantly reduced in *matα4-GAL-VP16/UAS-Rab5.S43N*; *mv*^{3}*/Df* embryos (n = 50). Scale bar, 100 μm. See also Figure S5.

(B) RAB5 (red), α-tubulin (green), and DAPI (blue) staining of control, CHS, and CHS RAB5.S43N fibroblasts in interphase and metaphase. CHS fibroblasts show aggregates of RAB5 and the phenotype is ameliorated after RAB5A.S43N expression. Scale bar, 50 μm.

requirement to relocate *Drosophila*'s NuMa-like protein, Mud, to the spindle poles (Capalbo et al., 2011). Expression of Rab5.S43N in otherwise wild-type embryos increased centrosomal recruitment of Msps (Figures 6A and 6C - black line) to a level higher than in control wild-type embryos (Figures 6A and 6C - gray line) and expression of Rab5.S43N in *mv*^{3}*/Df*-derived embryos restored the levels of Msps at centrosomes to levels comparable with those in control wild-type embryos (Figures 6A and 6C - blue line). Thus, not only is dominant-negative Rab5.S43N able to rescue vesicle size but also the centrosome association of Msps in *mv*-derived embryos. In similar experiments, we examined γ-tubulin levels at centrosomes during mitosis and also found its levels to be reduced in *mv*^{3}*/Df*-derived embryos compared with those in wild-type embryos and that

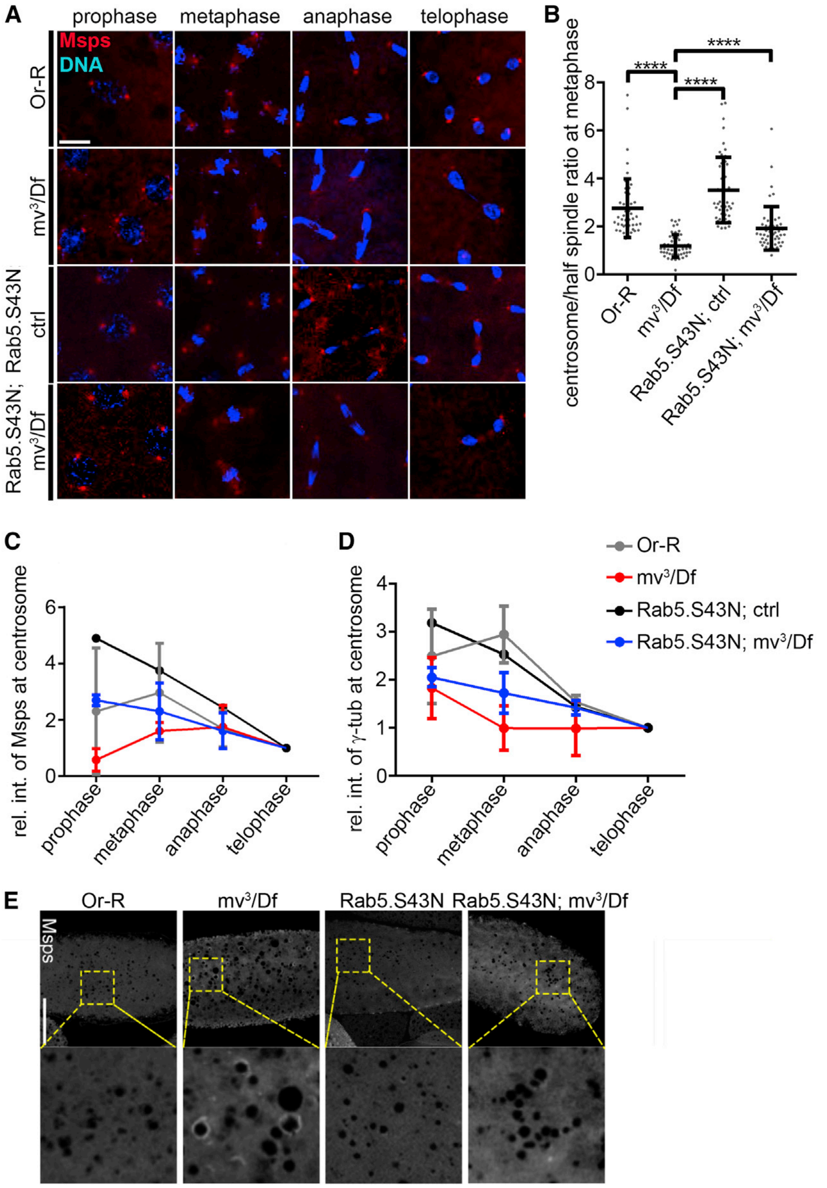

Figure 6. Mauve is required for the centrosomal association of Msps

(A) Different stages of embryonic nuclear division cycles stained to reveal Msps (red) and DNA (blue) in embryos derived from mothers of the indicated genotypes. Scale bar, 10 μm. See also Figure S6C.

(B) Quantitation of Msps immunostaining in metaphase spindles displayed as a ratio of the fluorescence intensity at the spindle pole to the corresponding half spindle (*Or-R*, 2.76 ± 0.17 n=50; *mv³/Df*, 1.176 ± 0.066 n = 50; *Rab5.S43N*, 3.506 ± 0.1943 n = 50; *Rab5.S43N mv³/Df*, 1.908 ± 0.1258 n = 52). Unpaired t test: ****$p < 0.0001$

(C) Quantitation of fluorescence intensity of Msps immunostaining at centrosomes in different phases of mitosis in embryos derived from mothers of the indicated genotypes. Values normalized to telophase values. Related to Figure S6C.

(D) Quantitation of fluorescence intensity of γ-tubulin immunostaining at centrosomes at indicated mitotic phases in embryos derived from mothers of the indicated genotypes. Values normalized to telophase values. Related to Figure S6D.

(E) Msps accumulate ectopically around enlarged LROs in *mauve* mutant embryos and this can be rescued after overexpression of Rab5.S43N in *mauve* mutant background driven by *P{matα4-GAL-VP16}*. Scale bar, 100 μm.

γ-tubulin levels could be rescued by overexpressing Rab5.S43N (Figures 6D and S6D). We also observed similar effects of *mv* upon the centrosomal association of the PCM protein, Centrosomin, but could not detect significant differences in centrosomal DPlp (Pericentrin-like protein) or Dspd-2 (spindle defective 2) (data not shown).

Although the total amount of Msps protein was not affected in *mv*-derived mutant embryos (Figure S6B), we found that it became re-distributed; Msps ectopically localized around the enlarged YGs correlating with its reduction in peri-centrosomal regions in mitosis (Figures 6A–6C, 6E and S6C). Moreover, the YG-associated Msps in *mv*-derived embryos was greatly reduced by Rab5.S43N (Figure 6E). As Msps and Rab5 are among many maternally provided proteins stored in LDs for use in early development (Cermelli et al., 2006), these findings together suggest that mutation in *mauve* results in a fusion between YGs and LDs leading to ectopic accumulation of Msps around YGs at the expense of its localization at the centrosome and so its availability for mitosis.

Mauve is required for effective MT nucleation by the spindle poles

The above results suggested that Mauve is required for centrosome maturation, which generates effective MTOCs in mitosis. To assess centrosomal MT nucleation activity in *mv³/Df*-derived and *mv^ros/Df*-derived (not shown) embryos, we carried out MT regrowth assays after cold-induced depolymerization of embryo MTs (Figures 7A, 7B, and S7). In all cases, a 0°C treatment was sufficient to completely depolymerize MTs (0 time). In wild-type embryos, MTs already began to be nucleated from centrosomes after 30 s of warming, increasing in density over time; they were also nucleated from chromatin after 3 min and could re-form normal-looking bipolar spindles by 5 min. By contrast, no MTs could be seen 30 s after shifting *mv*-derived embryos back to room temperature (Figure S7A). Even after 1 min, the density of MTs nucleated at centrosomes was much less than control wild-type embryos. We could, however, see chromatin nucleated MTs in *mv*-derived embryos after 1 min, a time at which MTs were not yet seen at chromatin in control embryos. This accords with the finding that removal of one MT nucleating system from the spindles of *Drosophila* embryos results in the amplification of synergistic MT nucleating activity

CellPress
OPEN ACCESS

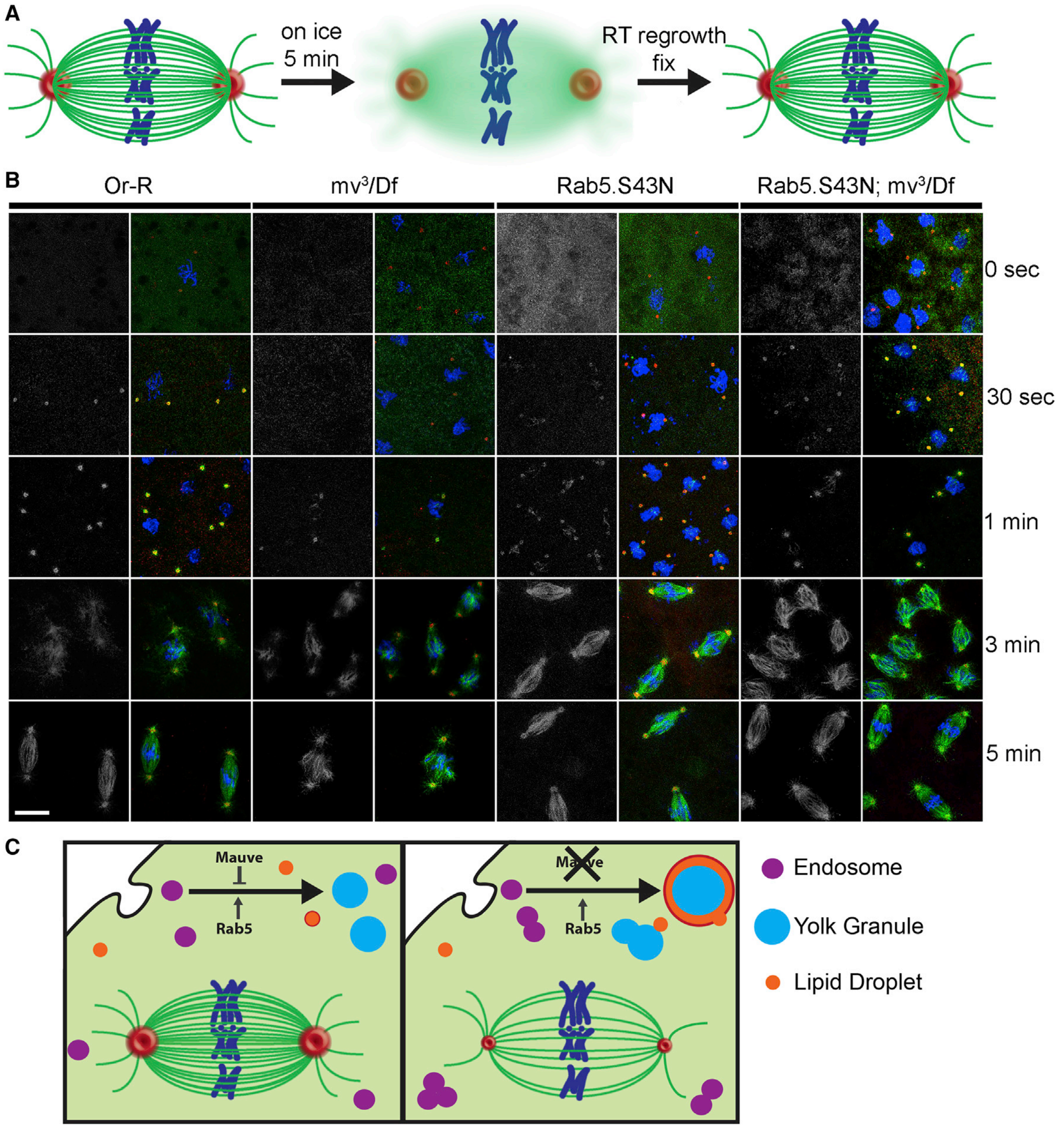

Figure 7. Mauve is required for effective MT nucleation by the spindle poles

(A) Scheme of MT regrowth. Living embryos were kept for 5 min at 0°C to depolymerize MTs and then shifted to room temperature to allow MT regrowth before fixing and staining at indicated times.

(B) Embryos derived from females of the indicated genotypes treated according to the regimen in (A) for the indicated times and stained to reveal α-tubulin, green; Centrosomin, red; and DNA (blue). MT regrowth from centrosomes is diminished in *mv*^{3}*/Df* and rescued by expression of Rab5.S43N. At least 10 different embryos were analyzed in metaphase for each genotype at each time point. Scale bar, 10 μm.

(C) Schematic of the antagonistic role of Mauve and Rab5 in *Drosophila* embryos. In wild-type embryos, YPs are taken up via endocytosis and stored in YGs. Mauve regulates vesicle fusion allowing the co-existence of discrete populations of endosomes, YGs and LDs within the same cytoplasm. When Mauve is depleted, an uncontrolled fusion between YGs and LDs leads to enlarged vesicles, aggregates of Rab5-positive endosomes, and an imbalance in the pool of centrosomal proteins. Msps (red) becomes sequestered in enlarged YGs at the expense of centrosomes, which exhibit reduced MT nucleation. These uncontrolled fusions can be counteracted by overexpression of dominant-negative Rab5.S43N. Vesicles, organelles, and cellular structures are not to scale.

(Hayward et al., 2014). After 3 min, the MTs nucleated from chromatin were much more prominent than those nucleated from centrosomes but even after 5 min, spindles were still incomplete (Figure S7B). Thus, MT regrowth kinetics following depolymerization suggest that the *mv* mutation leads to reduced MT nucleation from centrosomes.

As overexpression of Rab5.S43N increases Msps and γ-tubulin recruitment at centrosomes, we asked whether

dominant-negative Rab5 could restore regrowth of depolymerized MTs in *mv*-derived embryos. Indeed, Rab5.S43N led to a more rapid accumulation of MTs than in either the *mv* mutant or wild-type backgrounds such that complete spindles were already re-formed after 3 min of MT regrowth (Figure 7B). This supports our previous conclusion that the recruitment of PCM and MT minus-end associated molecules required for spindle pole MT nucleation are deficient in *mv*-derived embryos.

DISCUSSION

Our study has identified a requirement for Mauve, the *Drosophila* ortholog of the human LYST protein, in regulating LRO fusion and in the recruitment and/or maintenance of PCM at centrosomes in the syncytial embryo. Previous studies of *Drosophila mv* mutants suggested a role for Mauve in suppressing the homotypic fusion of LROs (Rahman et al., 2012). Here, we extend those observations by showing that Mauve also regulates heterotypic fusion between LROs and LDs and by showing that Mauve interacts with molecules that regulate the behavior of interphase and mitotic MTs. We also show that dominant-negative Rab5 not only rescues the LRO enlargement defect in *mv*-derived embryos but also ameliorates recruitment of Msps and PCM at centrosomes. The participation of LDs in LRO fusion that we now describe could have been previously overlooked because of the lower numbers of LDs in other tissues compared with those in embryos or through specific differences in the mutant alleles under study.

Our finding that high levels of Mauve did not induce the formation of smaller sized vesicles (Figures 1E, S1C, and S1D) together with our live imaging of excessive fusion events of autofluorescent vesicles during oogenesis in *mv* mutant females (Video S2) are consistent with a role for Mauve as a negative regulator of vesicle fusion. The behavior of LDs and the incorporation of their content into the dramatically enlarged YGs of *mv*-derived embryos (Figures 2H and 7C) is also consistent with this model.

Several lines of evidence support a role for *Drosophila* Mauve protein in regulating MT nucleation. First, we find an enrichment of Mv-mCherry around the spindle and centrosomes during mitosis (Figures 4C and S4A). Second, Mauve co-purifies with γ-tubulin and Msps (Figures 4D and S4C). Third, the rosario phenotype of *mauve*-derived embryos is enhanced by mutations in *d-tacc* or *msps*, suggesting co-involvement of Mauve and the D-TACC:Msps complex in establishing and/or maintaining the MT-mediated organization of the syncytium that ensures dividing nuclei are at the cortex and endoreduplicating yolk nuclei in the interior. Fourth, embryos derived from *mv* mutant mothers have reduced amounts of both Msps and γ-tubulin at centrosomes, in accord with the diminished MT nucleating capacity of these centrosomes. Fifth, in line with the reduced amounts of MT nucleating molecules at centrosomes, the regrowth of de-polymerized MTs from centrosomes is compromised in *mv*-derived embryos (Figure 7B).

Mauve's co-purification with Msps, but not its D-TACC partner protein, is another indicator that Msps can exist independently of D-TACC. Indeed, Msps is present in several separate pools: independent of D-TACC at the centrosome (Lee et al., 2001); in complex with the D-TACC: Clathrin complex on the spindle (Booth et al., 2011; Foraker et al., 2012; Fu et al., 2010; Lin et al., 2010); with the MT minus-end protein Patronin to assemble perinuclear non-centrosomal MTOCs (ncMTOCs) (Zheng et al., 2020); with the Augmin complex at kinetochores (Bucciarelli et al., 2009); and in complex with endosomal proteins such as Mauve. We speculate that mutations affecting the constitution of Msps complexes at any one of these sites can affect another.

Our finding of defects in mitotic MT nucleation by centrosomes in *mv*-derived embryos suggests that there might be similar requirements at later developmental stages that may have been overlooked because flies can progress through most of the development without functional centrosomes (Basto et al., 2006; Megraw et al., 2001).

The increased NUF seen in *mv*-derived embryos is likely to be a secondary consequence of disruption to either or both membrane trafficking and mitosis. NUF was first described for the mutant of the *nuf* gene encoding an ADP ribosylation factor effector that associates with Rab11. Nuf protein is required to organize recycling endosomes in the coordinated processes of membrane trafficking and actin remodeling and embryos deficient for Rab11 also show a strong NUF phenotype (Cao et al., 2008; Rothwell et al., 1999; Hickson et al., 2003; Riggs et al., 2007). Together this suggests the possibility that NUF in *mv* mutants could result from the accumulation of endosomal components in the enlarged YGs, which would diminish numbers of recycling endosomes and their associated Rab11-Nuf complex. NUF can also occur as a Chk2 protein kinase-mediated response to DNA damage (DSBs), activated by DNA lesions at mitotic onset (Takada et al., 2003). However, we found no evidence for DNA damage marked by the accumulation of phosphorylated γ-H2Av at DSBs (Figure S3C). Finally, NUF also occurs in response to a wide range of primary or secondary mitotic defects. Indeed, failure of the sequestration of histone H2Av to LDs results in embryos that display mitotic defects, nuclear fallout, and reduced viability (Li et al., 2014).

Dominant-negative Rab5 suppresses enlarged YG formation and the mitotic defects of *mv*-derived embryos in accord with known roles of Rab5 at the early endosome and growing indications of a requirement for Rab5 in mitosis (Capalbo et al., 2011; Lanzetti, 2012; Serio et al., 2011). Rab5 also mediates transient interactions between LDs and early endosomes that enable the transport of lipids between the two without resulting in their fusion (Liu et al., 2007). We cannot rule out the possibility that Msps transiently localizes to LROs in wild-type embryos because we observed LD-YG associations in wild-type embryos and Msps is a component of LDs (Cermelli et al., 2006). The incorporation of Msps and LD markers into the enlarged YGs in *mv*-derived embryos is also rescued by a dominant-negative form of Rab5 and reciprocally, levels of Msps at centrosomes are restored. This suggests that mutation in *mauve* leads to mislocalization of Msps around YGs at the expense of its localization at the centrosome and so its availability for mitosis. Suppression of these *mv* phenotypes by dominant-negative Rab5 could therefore either reflect a passive restoration of the balance of Msps between YGs and spindle poles once YG fusion is prevented or a more active role of Rab5 in organizing the spindle poles.

Our findings add to a small but growing body of evidence for the roles of endocytic membrane trafficking in regulating

centrosomal function (Naslavsky and Caplan, 2020). To our knowledge, there are no reports of a membrane-independent role of Rab5 although other groups have reported examples of trafficking proteins involved in MT nucleation in a membrane-independent manner, such as ALIX, a PCM component in human and fly cells, whose recruitment depends on Cnn/Cep215 and D-Spd2/Cep192 (Malerød et al., 2018). The late endosome marker Rab11 also appears to be a part of a dynein-dependent retrograde transport pathway bringing MT nucleating factors and spindle pole proteins to mitotic spindle poles (Das et al., 2014; Hehnly and Doxsey, 2014). It is not clear whether Rab5-associated structures mature to Rab11-associated structures in mitosis as they do in interphase but it seems that the two vesicle types might have overlapping functions at centrosomes in mitosis. It will be of future interest to put our current findings into context with these earlier demonstrations of roles of Rab5- and Rab11-containing endosomes in spindle function.

The dynamic relationship between endosomal trafficking and recruitment of MT nucleating molecules onto centrosomes may all have relevance for the role of LYST at the IS and how this is affected in CHS. Thus, it is conceivable that there may be a convergence of the two functions of the LYST protein in lymphocytes, both in regulating the size of LROs and in facilitating the correct positing of centrosomes and membraneous structures. Further studies will be required to clarify the precise roles of LYST in regulating vesicle trafficking and MT nucleation in this particular cell type.

Limitations of the study

Although our results strongly indicate Mauve to act as a negative regulator of vesicle fusion, we did not directly assess the fusion ability of LROs. In part, this was limited by the autofluorescent nature of YGs and LDs that restricted the extent to which we could use fluorescently tagged proteins to visualize membrane components of these bodies in dynamic studies. Future work should aim to complement our findings in cell culture and in cell-free systems to determine whether the involvement of both LROs and LDs is widespread. In a similar vein, it will be important to assess whether the roles of LYST proteins in regulating MT dynamics are conserved as implied by our findings. This would require carrying out studies of MT dynamics in other cell types, particularly in mammalian cells.

STAR★METHODS

Detailed methods are provided in the online version of this paper and include the following:

- KEY RESOURCES TABLE
- RESOURCE AVAILABILITY
  - Lead contact
  - Materials availability
  - Data and code availability
- EXPERIMENTAL MODEL AND SUBJECT DETAILS
  - Fly lines and genetics
  - Cell lines
- METHODS DETAILS
  - Cytogenetic mapping of mv alleles
  - Cloning
  - qPCR
  - Immunofluorescence and live imaging
  - Antibodies
  - Mass spectrometry, Co-IPs, Coomassie staining and Western blots
  - Illustrations and figure preparation
- QUANTITATIONS AND STATISTICAL ANALYSIS

SUPPLEMENTAL INFORMATION

Supplemental information can be found online at https://doi.org/10.1016/j.devcel.2021.02.019.

ACKNOWLEDGMENTS

We are grateful to James Mason (NIEHS), Hiro Ohkura (Edinburgh), and Jordan Raff (Oxford) for kindly providing reagents. We thank the University of Iowa DSHB, the BACPAC Resource Center (BPRC), NCI Frederick National Laboratory for Cancer Research, Coriell Institute for Medical Research and Addgene for reagents; Bloomington Drosophila Stock Center (BDSC), Drosophila Transgenesis Facility, CMB, (Spain), and the Fly Facility of the Department of Genetics (Cambridge) for fly stocks; IBB PAS (Warsaw) for mass spectrometry; and members of the Glover lab for fruitful discussion. We are grateful for grant support from Wellcome (RG84496) and NIH (R01NS119614) to D.M.G.

AUTHOR CONTRIBUTIONS

R.L. designed and conducted the experiments and prepared all figures. H.R. generated the anti-Msps antibody. S.L. discovered *mv^{ros}* and initiated its cytological mapping and characterization of its MEL phenotype. D.M.G. supervised the project, raised funding, and wrote the manuscript with R.L.

DECLARATION OF INTERESTS

The authors declare no competing interests.

Received: March 23, 2020
Revised: November 17, 2020
Accepted: February 17, 2021
Published: March 15, 2021

REFERENCES

Barbosa, M.D.F.S., Nguyen, Q.A., Tchernev, V.T., Ashley, J.A., Detter, J.C., Blaydes, S.M., Brandt, S.J., Chotai, D., Hodgman, C., Solari, R.C.E., et al. (1996). Identification of the homologous beige and Chediak–Higashi syndrome genes. Nature *382*, 262–265.

Basto, R., Lau, J., Vinogradova, T., Gardiol, A., Woods, C.G., Khodjakov, A., and Raff, J.W. (2006). Flies without centrioles. Cell *125*, 1375–1386.

Bettencourt-Dias, M., Rodrigues-Martins, A., Carpenter, L., Riparbelli, M., Lehmann, L., Gatt, M.K., Carmo, N., Balloux, F., Callaini, G., and Glover, D.M. (2005). SAK/PLK4 is required for centriole duplication and flagella development. Curr. Biol. *15*, 2199–2207.

Booth, D.G., Hood, F.E., Prior, I.A., and Royle, S.J. (2011). A TACC3/ch-TOG/clathrin complex stabilises kinetochore fibres by inter-microtubule bridging. EMBO J. *30*, 906–919.

Bownes, M., and Hames, B.D. (1977). Accumulation and degradation of three major yolk proteins in Drosophila melanogaster. J. Exp. Zool. *200*, 149–156.

Boxer, L.A., Albertini, D.F., Baehner, R.L., and Oliver, J.M. (1979). Impaired microtubule assembly and polymorphonuclear leucocyte function in the Chediak-Higashi syndrome correctable by ascorbic acid. Br. J. Haematol. *43*, 207–213.

Brennan, M.D., Weiner, A.J., Goralski, T.J., and Mahowald, A.P. (1982). The follicle cells are a major site of vitellogenin synthesis in Drosophila melanogaster. Dev. Biol. *89*, 225–236.

Bucciarelli, E., Pellacani, C., Naim, V., Palena, A., Gatti, M., and Somma, M.P. (2009). Drosophila Dgt6 Interacts with Ndc80, Msps/XMAP215, and γ-tubulin to promote kinetochore-driven MT formation. Curr. Biol. *19*, 1839–1845.

Cao, J., Albertson, R., Riggs, B., Field, C.M., and Sullivan, W. (2008). Nuf, a Rab11 effector, maintains cytokinetic furrow integrity by promoting local actin polymerization. J Cell Biol *182*, 301–313.

Capalbo, L., D'Avino, P.P., Archambault, V., and Glover, D.M. (2011). Rab5 GTPase controls chromosome alignment through lamin disassembly and relocation of the NuMA-like protein Mud to the poles during mitosis. Proc. Natl. Acad. Sci. USA *108*, 17343–17348.

Cermelli, S., Guo, Y., Gross, S.P., and Welte, M.A. (2006). The lipid-droplet proteome reveals that droplets are a protein-storage depot. Curr. Biol. *16*, 1783–1795.

Chiang, S.C.C., Wood, S.M., Tesi, B., Akar, H.H., Al-Herz, W., Ammann, S., Belen, F.B., Caliskan, U., Kaya, Z., Lehmberg, K., et al. (2017). Differences in granule morphology yet equally impaired exocytosis among cytotoxic T cells and NK cells from Chediak-Higashi syndrome patients. Front. Immunol. *8*, 426.

Cullen, C.F., Deák, P., Glover, D.M., and Ohkura, H. (1999). mini spindles: a gene encoding a conserved microtubule-associated protein required for the integrity of the mitotic spindle in Drosophila. J. Cell Biol. *146*, 1005–1018.

Das, S., Hehnly, H., and Doxsey, S. (2014). A new role for Rab GTPases during early mitotic stages. Small GTPases *5*, e29565.

DiMario, P.J., and Mahowald, A.P. (1987). Female sterile (1) yolkless: a recessive female sterile mutation in Drosophila melanogaster with depressed numbers of coated pits and coated vesicles within the developing oocytes. J. Cell Biol. *105*, 199–206.

Durchfort, N., Verhoef, S., Vaughn, M.B., Shrestha, R., Adam, D., Kaplan, J., and Ward, D.M. (2012). The enlarged lysosomes in beigej cells result from decreased lysosome fission and not increased lysosome fusion. Traffic *13*, 108–119.

Fagotto, F. (1995). Regulation of yolk degradation, or how to make sleepy lysosomes. J. Cell Sci. *108*, 3645–3647.

Foe, V.E., and Alberts, B.M. (1983). Studies of nuclear and cytoplasmic behaviour during the five mitotic cycles that precede gastrulation in Drosophila embryogenesis. J. Cell Sci. *61*, 31–70.

Foraker, A.B., Camus, S.M., Evans, T.M., Majeed, S.R., Chen, C.Y., Taner, S.B., Corrêa, I.R., Doxsey, S.J., and Brodsky, F.M. (2012). Clathrin promotes centrosome integrity in early mitosis through stabilization of centrosomal ch-TOG. J. Cell Biol. *198*, 591–605.

Frankel, F.R., Tucker, R.W., Bruce, J., and Stenberg, R. (1978). Fibroblasts and macrophages of mice with the Chediak-Higashi-like syndrome have microtubules and actin cables. J. Cell Biol. *79*, 401–408.

Fu, W., Tao, W., Zheng, P., Fu, J., Bian, M., Jiang, Q., Clarke, P.R., and Zhang, C. (2010). Clathrin recruits phosphorylated TACC3 to spindle poles for bipolar spindle assembly and chromosome alignment. J. Cell Sci. *123*, 3645–3651.

Gergely, F., Kidd, D., Jeffers, K., Wakefield, J.G., and Raff, J.W. (2000). D-TACC: a novel centrosomal protein required for normal spindle function in the early Drosophila embryo. EMBO J. *19*, 241–252.

Gil-Krzewska, A., Saeed, M.B., Oszmiana, A., Fischer, E.R., Lagrue, K., Gahl, W.A., Introne, W.J., Coligan, J.E., Davis, D.M., and Krzewski, K. (2018). An actin cytoskeletal barrier inhibits lytic granule release from natural killer cells in patients with Chediak-Higashi syndrome. J. Allergy Clin. Immunol. *142*, 914–927.e6.

Gil-Krzewska, A., Wood, S.M., Murakami, Y., Nguyen, V., Chiang, S.C.C., Cullinane, A.R., Peruzzi, G., Gahl, W.A., Coligan, J.E., Introne, W.J., et al. (2016). Chediak-Higashi syndrome: lysosomal trafficking regulator domains regulate exocytosis of lytic granules but not cytokine secretion by natural killer cells. J. Allergy Clin. Immunol. *137*, 1165–1177.

Gillingham, A.K., Sinka, R., Torres, I.L., Lilley, K.S., and Munro, S. (2014). Toward a comprehensive map of the effectors of Rab GTPases. Dev. Cell *31*, 358–373.

Girard, E., Chmiest, D., Fournier, N., Johannes, L., Paul, J.L., Vedie, B., and Lamaze, C. (2014). Rab7 is functionally required for selective cargo sorting at the early endosome. Traffic *15*, 309–326.

Hayward, D., Metz, J., Pellacani, C., and Wakefield, J.G. (2014). Synergy between multiple microtubule-generating pathways confers robustness to centrosome-driven mitotic spindle formation. Dev. Cell *28*, 81–93.

Hehnly, H., and Doxsey, S. (2014). Rab11 endosomes contribute to mitotic spindle organization and orientation. Dev. Cell *28*, 497–507.

Hickson, G.R.X., Matheson, J., Riggs, B., Maier, V.H., Fielding, A.B., Prekeris, R., Sullivan, W., Barr, F.A., and Gould, G.W. (2003). Arfophilins are dual Arf/Rab 11 binding proteins that regulate recycling endosome distribution and are related to Drosophila nuclear fallout. Mol. Biol. Cell *14*, 2908–2920.

Kaplan, J., De Domenico, I., and Ward, D.M. (2008). Chediak-Higashi syndrome. Curr. Opin. Hematol. *15*, 22–29.

Kasravi, A., Walter, M.F., Brand, S., Mason, J.M., and Biessmann, H. (1999). Molecular cloning and tissue-specific expression of the mutator2 gene (mu2) in Drosophila melanogaster. Genetics *152*, 1025–1035.

Kühnlein, R.P. (2012). Thematic review series: lipid droplet synthesis and metabolism: from yeast to man. Lipid droplet-based storage fat metabolism in Drosophila. J. Lipid Res. *53*, 1430–1436.

Kypri, E., Schmauch, C., Maniak, M., and Lozanne, A. De. (2007). The BEACH protein LvsB is localized on lysosomes and postlysosomes and limits their fusion with early endosomes. Traffic *8*, 774–783.

Lanzetti, L. (2012). A novel function of Rab5 in mitosis. Small GTPases *3*, 168–172.

Lee, M.J., Gergely, F., Jeffers, K., Peak-Chew, S.Y., and Raff, J.W. (2001). Msps/XMAP215 interacts with the centrosomal protein D-TACC to regulate microtubule behaviour. Nat. Cell Biol. *3*, 643–649.

Li, Z., Johnson, M.R., Ke, Z., Chen, L., and Welte, M.A. (2014). Drosophila lipid droplets buffer the H2Av supply to protect early embryonic development. Curr. Biol. *24*, 1485–1491.

Lin, C.H., Hu, C.K., and Shih, H.M. (2010). Clathrin heavy chain mediates TACC3 targeting to mitotic spindles to ensure spindle stability. J. Cell Biol. *189*, 1097–1105.

Liu, G., Sanghavi, P., Bollinger, K.E., Perry, L., Marshall, B., Roon, P., Tanaka, T., Nakamura, A., and Gonsalvez, G.B. (2015). Efficient endocytic uptake and maturation in Drosophila oocytes requires dynamitin/p50. Genetics *201*, 631–649.

Liu, P., Bartz, R., Zehmer, J.K., Ying, Y.S., Zhu, M., Serrero, G., and Anderson, R.G. (2007). Rab-regulated interaction of early endosomes with lipid droplets. Biochim. Biophys. Acta *1773*, 784–793.

Malerød, L., Le Borgne, R., Lie-Jensen, A., Eikenes, Å.H., Brech, A., Liestøl, K., Stenmark, H., and Haglund, K. (2018). Centrosomal ALIX regulates mitotic spindle orientation by modulating astral microtubule dynamics. EMBO J. *37*, e97741.

Marks, M.S., Heijnen, H.F.G.G., and Raposo, G. (2013). Lysosome-related organelles: unusual compartments become mainstream. Curr. Opin. Cell Biol. *25*, 495–505.

Megraw, T.L., Kao, L.R., and Kaufman, T.C. (2001). Zygotic development without functional mitotic centrosomes. Curr. Biol. *11*, 116–120.

Naslavsky, N., and Caplan, S. (2020). Endocytic membrane trafficking in the control of centrosome function. Curr. Opin. Cell Biol. *65*, 150–155.

Oliver, C., and Essner, E. (1975). Formation of anomalous lysosomes in monocytes, neutrophils, and eosinophils from bone marrow of mice with Chédiak-Higashi syndrome. Lab. Invest. *32*, 17–27.

Oliver, J.M., and Zurier, R.B. (1976). Correction of characteristic abnormalities of microtubule function and granule morphology in Chediak-Higashi syndrome with cholinergic agonists. J. Clin. Invest. *57*, 1239–1247.

Ostlund, R.E., Tucker, R.W., Leung, J.T., Okun, N., and Williamson, J.R. (1980). The cytoskeleton in Chediak-Higashi syndrome fibroblasts. Blood *56*, 806–811.

Perou, C.M., Leslie, J.D., Green, W., Li, L., Ward, D.M., and Kaplan, J. (1997). The beige/Chediak-Higashi syndrome gene encodes a widely expressed cytosolic protein. J. Biol. Chem. *272*, 29790–29794.

Pryzwansky, K.B., Schliwa, M., and Boxer, L.A. (1985). Microtubule organization of unstimulated and stimulated adherent human neutrophils in Chediak-Higashi syndrome. Blood *66*, 1398–1403.

Rahman, M., Haberman, A., Tracy, C., Ray, S., and Krämer, H. (2012). Drosophila mauve mutants reveal a role of LYST homologs late in the maturation of phagosomes and autophagosomes. Traffic *13*, 1680–1692.

Riggs, B., Fasulo, B., Royou, A., Mische, S., Cao, J., Hays, T.S., and Sullivan, W. (2007). The concentration of Nuf, a Rab11 effector, at the microtubule-organizing center is cell cycle regulated, dynein-dependent, and coincides with furrow formation. Mol. Biol. Cell *18*, 3313–3322.

Riparbelli, M.G., and Callaini, G. (2003). Assembly of yolk spindles in the early Drosophila embryo. Mech. Dev. *120*, 441–454.

Rodrigues-Martins, A., Riparbelli, M., Callaini, G., Glover, D.M., and Bettencourt-Dias, M. (2007). Revisiting the role of the mother centriole in centriole biogenesis. Science *316*, 1046–1050.

Rothwell, W.F., Zhang, C.X., Zelano, C., Hsieh, T.S., and Sullivan, W. (1999). The Drosophila centrosomal protein Nuf is required for recruiting Dah, a membrane associated protein, to furrows in the early embryo. J. Cell Sci. *112*, 2885–2893.

Schindelin, J., Arganda-Carreras, I., Frise, E., Kaynig, V., Longair, M., Pietzsch, T., Preibisch, S., Rueden, C., Saalfeld, S., and Schmid, B. (2012). Fiji: an open-source platform for biological-image analysis. Nat. Methods. *9*, 676–682.

Serio, G., Margaria, V., Jensen, S., Oldani, A., Bartek, J., Bussolino, F., and Lanzetti, L. (2011). Small GTPase Rab5 participates in chromosome congression and regulates localization of the centromere-associated protein CENP-F to kinetochores. Proc. Natl. Acad. Sci. USA *108*, 17337–17342.

Sliter, T.J., Henrich, V.C., Tucker, R.L., and Gilbert, L.I. (1989). The genetics of the Dras3-roughened-ecdysoneless chromosomal region (62B3-4 to 62D3-4) in Drosophila melanogaster: analysis of recessive lethal mutations. Genetics *123*, 327–336.

Stenmark, H., Parton, R.G., Steele-Mortimer, O., Lütcke, A., Gruenberg, J., and Zerial, M. (1994). Inhibition of rab5 GTPase activity stimulates membrane fusion in endocytosis. EMBO J. *13*, 1287–1296.

Takada, S., Kelkar, A., and Theurkauf, W.E. (2003). Drosophila checkpoint kinase 2 couples centrosome function and spindle assembly to genomic integrity. Cell *113*, 87–99.

Tsuruhara, T., Koenig, J.H., and Ikeda, K. (1990). Synchronized endocytosis studied in the oocyte of a temperature-sensitive mutant of Drosophila melanogaster. Cell Tissue Res. *259*, 199–207.

Vanlandingham, P.A., and Ceresa, B.P. (2009). Rab7 regulates late endocytic trafficking downstream of multivesicular body biogenesis and cargo sequestration. J. Biol. Chem. *284*, 12110–12124.

Venken, K.J.T., Carlson, J.W., Schulze, K.L., Pan, H., He, Y., Spokony, R., Wan, K.H., Koriabine, M., de Jong, P.J., White, K.P., et al. (2009). Versatile P [acman] BAC libraries for transgenesis studies in Drosophila melanogaster. Nat. Methods *6*, 431–434.

Ward, D.M., Griffiths, G.M., Stinchcombe, J.C., and Kaplan, J. (2000). Analysis of the lysosomal storage disease Chediak-Higashi syndrome. Traffic *1*, 816–822.

Warren, T.G., and Mahowald, A.P. (1979). Isolation and partial chemical characterization of the three major yolk polypeptides from Drosophila melanogaster. Dev. Biol. *68*, 130–139.

Willingham, M.C., Spicer, S.S., and Vincent, R.A. (1981). The origin and fate of large dense bodies in beige mouse fibroblasts. Lysosomal fusion and exocytosis. Exp. Cell Res. *136*, 157–168.

Zheng, Y., Buchwalter, R.A., Zheng, C., Wight, E.M., Chen, J.V., and Megraw, T.L. (2020). A perinuclear microtubule-organizing centre controls nuclear positioning and basement membrane secretion. Nat. Cell Biol. *22*, 297–309.

STAR★METHODS

KEY RESOURCES TABLE

REAGENT or RESOURCE	SOURCE	IDENTIFIER
**Antibodies**		
Anti-α-Tubulin antibody, Mouse monoclonal clone DM1A, purified from hybridoma cell culture	Sigma-Aldrich	Cat# T6199; RRID: AB_477583
Anti-Msps rb 2219, rabbit polyclonal serum against the aa 1350-1785	Hélène Rangone, this manuscript	rb 2219
Anti-Cnn, rabbit polyclonal	Bettencourt-Dias et al., 2005	rb 7647
Anti-Dplp, chicken polyclonal	Rodrigues-Martins et al., 2007	N/A
Anti-mCherry antibody	Abcam	Cat# ab167453; RRID: AB_2571870
Anti-LAMP-1 (human), clone H4A3, mouse monoclonal	DSHB	Cat# H4A3; RRID: AB_2296838
Anti-γ-Tubulin antibody, clone GTU-88, mouse monoclonal, purified from hybridoma cell culture	Sigma-Aldrich	Cat# T5326; RRID: AB_532292
Anti-p55/DCAF1 antibody—ChIP Grade, rabbit polyclonal	Abcam	Cat# ab1766; RRID: AB_302606
Anti-Rab5 antibody—Drosophila Early Endosome Marker	Abcam	Cat# ab31261; RRID: AB_882240
Recombinant Anti-Rab5 antibody [EPR21801]	Abcam	ab218624
Histone H2AvD pS137 Antibody	Rockland	Cat# 600-401-914; RRID: AB_828383
RFP-Booster Alexa Fluor® 568	Chromotek	rb2AF568
**Bacterial and virus strains**		
One Shot™ TOP10 Chemically Competent *E. coli*	Thermo Fisher Scientific	Cat#C404006
One Shot™ *ccd*B Survival™ 2 T1^R Competent Cells	Thermo Fisher Scientific	Cat#A10460
One Shot™ BL21(DE3) Chemically Competent *E. coli*	Thermo Fisher Scientific	Cat#C600003
E.coli SW102 strain	The NCI at Frederick and the Frederick National Laboratory for Cancer Research	SW102
TransforMax™ EPI300™ Electrocompetent *E. coli*	Epicentre - Cambio	EC300110
**Chemicals, peptides, and recombinant proteins**		
Vector Vectashield Mounting Media containing DAPI	Vector Laboratories	H-1200
Nile Red, lipophilic stain	Abcam	ab219403
G-418 Solution	Merck	4727878001
FuGENE® HD Transfection Reagent	Promega	E2311
Guanosine 5'-diphosphate [GDP] disodium salt, Nucleoside diphosphate	Abcam	ab146529
GppNHp, Non-hydrolyzable GTP analog	Abcam	ab146659
Calf Intestinal Alkaline Phosphatase (CIP)	NEB	M0290
T4 DNA Ligase	NEB	M0202S
**Critical commercial assays**		
Gateway™ BP Clonase™ II Enzyme mix	Thermo Fisher Scientific	11789020
Gateway™ LR Clonase™ II Enzyme mix	Thermo Fisher Scientific	11791020

(*Continued on next page*)

CellPress
OPEN ACCESS

Continued

REAGENT or RESOURCE	SOURCE	IDENTIFIER
Gibson Assembly Cloning Kit	NEB	E5510S
RFP-Trap Magnetic Agarose	Chromotek	Cat# rtma-20; RRID: AB_2631363
GFP-Trap Magnetic Agarose	Chromotek	Cat# gtma-20,;RRID: AB_2631358
Ni-NTA Agarose beads	Qiagen	ID: 30210
BACMAX™ DNA Purification Kit	Epicentre - Cambio	N/A
RNeasy mini kit	Qiagen	ID: 74104
SuperScript™ III First-Strand Synthesis System	Thermo Fisher Scientific	18080400
Power SYBR® Green RNA-to-CT™ 1-Step Kit	Thermo Fisher Scientific	4391178
**Experimental models: cell lines**		
Human untrasformed fibroblasts CHEDIAK-HIGASHI SYNDROME	Coriell Institute for Medical Research	Cat# GM02075; RRID: CVCL_CW70 (CHS in this manuscript)
Human untrasformed fibroblasts CHEDIAK-HIGASHI SYNDROME stable expressing human EGFP-RAB5A.S43N	This manuscript	NA (CHS RAB5.S43N in this manuscript)
Human untrasformed fibroblasts from skin	Coriell Institute for Medical Research	Cat# AG21862; RRID: CVCL_2Y59
**Experimental models: organisms/strains**		
D.melanogaster Oregon-R (wild-type)	Fly Facility at Department of Genetics (University of Cambridge)	*Or-R*
D. melanogaster l(3)dre6/TM6B (renamed as mv^3*/TM6B)*	Sliter et al., 1989	mv^3*/TM6B*
D. melanogaster fs(3)ros/TM6B (renamed as mv^{ros}*/TM6B)*	Salud Llamazares	mv^{ros}*/TM6B*
D. melanogaster In(3LR)264, mv^1*/TM6B*	Bloomington Drosophila Stock Center	Cat# 1222; RRID: BDSC_1222
D. melanogaster Df(3L)Aprt66/TM6B	Prof. James M. Mason (University of California and NIEHS)	N/A
D. melanogaster Df(3L)Aprt-198	Prof. James M. Mason (University of California and NIEHS)	N/A
D. melanogaster Df(3L)R-G7, rho^{ve-1}*/TM6B*	Bloomington Drosophila Stock Center	Cat# 2400; RRID: BDSC_2400 (Df in this manuscript)
D.melanogaster w^{1118}*; Df(3L) BSC366/TM6C*	Bloomington Drosophila Stock Center	Cat# 24390; RRID: BDSC_24390
D. melanogaster w^{1118}*; Df(3L)ED4284, P {3'.RS5+3.3'}ED4284/TM6C*	Bloomington Drosophila Stock Center	Cat# 8056; RRID: BDSC_8056
D. melanogaster w1118; Df(3R)Exel6142, P {XP-U}Exel6142/TM6B, Tb1	Bloomington Drosophila Stock Center	Cat# 7621; RRID: BDSC_7621 (Df-TACC in this manuscript)
D.melanogaster Ub-Tubulin-RFP, Ub-Fzr-GFP (Chr 2)	Prof. Jordan Raff (University of Oxford)	N/A
D.melanogaster Ub-Msps-GFP (Chr 2)	Prof. Jordan Raff (University of Oxford)	N/A
D. melanogaster $TACC^1$*/TM6B*	Prof. Jordan Raff (University of Oxford)	N/A
D. melanogaster $msps^{MJ208}$*/TM6B*	Prof. Hiro Ohkura (University of Edinburgh)	N/A
D. melanogaster Mv-mCherry/SM6A	this manuscript	*Mv-mCherry/SM6A*
D. melanogaster Mv-FLAG/SM6A (Mv-TC-FLAG)	this manuscript	*Mv-TC-FLAG/SM6A*
D. melanogaster UAS-Mv-GFP/SM6A	this manuscript	*UAS-Mv-GFP/SM6A*
D. melanogaster y^1 *w* P{UASp-YFP.Rab7.T22N}CG1578*^{19}	Bloomington Drosophila Stock Center	Cat# 23235; RRID: BDSC_23235
D. melanogaster y^1 *w*; P{UASp-YFP.Rab5.Q88L}Reph*^{24}	Bloomington Drosophila Stock Center	Cat# 9774; RRID: BDSC_9774

(*Continued on next page*)

Continued

REAGENT or RESOURCE	SOURCE	IDENTIFIER
D. melanogaster y[1] w; P{UASp-YFP.Rab7.Q67L}19*	Bloomington Drosophila Stock Center	Cat# 24103; RRID: BDSC_24103
D. melanogaster w; P{UAS-Rab5.S43N}2*	Bloomington Drosophila Stock Center	Cat# 42703; RRID: BDSC_42703
D. melanogaster P{matα4-GAL-VP16}V2H	Bloomington Drosophila Stock Center	Cat# 7062; RRID: BDSC_7062
D. melanogaster y1 w; P{UASp-YFP.Rab5.S43N}Eip75B02/TM3, Sb1*	Bloomington Drosophila Stock Center	Cat# 9772; RRID: BDSC_9772
D. melanogaster w1118; TI{TI}Rab5EYFP	Bloomington Drosophila Stock Center	Cat# 62543; RRID: BDSC_62543
Oligonucleotides		
Primers	See Table S1 for primers list	N/A
Recombinant DNA		
BAC clone Mv_TC_FLAG	this manuscript	Mv_TC_FLAG PBac{CH322-23O09}
BAC clone Mv_mCherry	this manuscript	Mv-mCherry PBac{CH322-23O09}
Empty entry plasmid pPWG-attB	this manuscript	pPWG-attB
clone UASp-Mv-GFP in pPWG-attB	this manuscript	UAS-Mv-GFP in pPWG-attB
clone Msps 1350-1785 aa in pDONR221	this manuscript	N/A
clone Msps 1350-1785 aa in pDEST17	this manuscript	N/A
Human EGFP-Rab5A.S34N	Addgene	RRID: Addgene_28045
BAC clone CH322-23O09	BACPAC Resources Center – BPRC	PBac{CH322-23O09}, attB-P[acman]-CmR-BW vector
Software and algorithms		
Fiji	Schindelin et al., 2012	RRID: SCR_002285
Adobe Illustrator Software	Adobe	RRID: SCR_010279
Adobe Photoshop Software	Adobe	RRID: SCR_014199
GraphPad Prism	GraphPad Software	RRID: SCR_002798
Huygens Software	Scientific Volume Imaging (SVI)	RRID: SCR_014237
Volocity	Quorum Technologie	RRID: SCR_002668

RESOURCE AVAILABILITY

Lead contact

Further information and requests for resources and reagents should be directed to and will be fulfilled by the Lead Contact, Ramona Lattao (rl489@cam.ac.uk) or David Glover (dmglover@caltech.edu).

Materials availability

All unique/stable reagents generated in this study are available from the Lead Contact without restriction.

Data and code availability

The published article includes all mass spectrometry data generated during this study.

EXPERIMENTAL MODEL AND SUBJECT DETAILS

Fly lines and genetics

All stocks were maintained at 25°C in standard media. The *mauve* allele *mv*^{3} was isolated as *l(3)dre6* line generated by ethyl methanesulfonate (EMS) and γ-ray mutagenesis of the *Dras3-Roughened-Ecdysoneless* chromosomal region (62B3-4 to 62D3-4) (Sliter et al., 1989). The lethal phenotype of *l(3)dre6* is due to an unknown second-site mutation. Our sequencing *of mauve* in *l(3)dre6/Df(3L) R-G7* identified a G to A point mutation in nucleotide position 3L: 1,952,183 that corresponds to the splicing acceptor site of intron 15-16. The *mauve* allele *mv*^{ros} was isolated by one of us (SL). In *fs(3)ros/Df(3L)R-G7* we were unable to sequence the region between 3L: 1,961,478 and 3L:1,959,258 likely due to complex rearrangements in the DNA of this region. The *mv*^{1} allele was obtained from the Bloomington Drosophila Stock Center (BDSC 1222), *In(*3LR*)264*, *mv*^{1}*/TM6B*: the inversion breakpoints localize in the *mauve* and *tacc* genes and result in a fusion protein connecting codons 1-2772 of mv with nucleotide 853 of isoform 4 of tacc (Rahman et al., 2012).

The deficiencies *Df(3L)Aprt66/TM6B* and *Df(3L)Aprt-198* were a kind gift from Prof. James M. Mason (University of California and NIEHS); *Df(3L)R-G7*, *rho^{ve-1}/TM6B* (BDSC 2400), *w^{1118}*; *Df(3L)BSC366/TM6C* (BDSC 24390), *w^{1118}*; *Df(3L)ED4284*, *P{3'.RS5+3.3'}ED4284/TM6C* (BDSC 8056), *w1118*; *Df(3R)Exel6142*, *P{XP-U}Exel6142/TM6B*, *Tb1 (BDSC 7621*, *Df TACC)* were obtained from the Bloomington Drosophila Stock Center (BDSC). Unless otherwise stated, all the experiments shown were carried out with *mv^3/Df(3L)R-G7*.

The *Ub-Tubulin-RFP*, *Ub-Fzr-GFP*, and *tacc^1/TM6B* lines were kindly provided by Prof Jordan W. Raff (University of Oxford). *msps^{MJ208}/TM6B* was a gift from Prof. Hiro Ohkura (University of Edinburgh).

The transgenic lines *Mv-mCherry and Mv-TC_FLAG* were generated by injecting the plasmid into a *y w M(eGFP*, *vas-int*, *dmRFP) ZH-2A*; *P{CaryP}attP40* stock (Fly Facility – Department of Genetics, University of Cambridge) whereas *UAS-Mv-GFP* was injected into *y1 M{vas-int.Dm}ZH-2A w**; *M{3xP3-RFP.attP'}ZH-22A* (*Drosophila* Transgenesis Facility, CMB, Spain).

Rab alleles were obtained from the Bloomington Drosophila Stock Center: y^1 w* P{UASp-YFP.Rab7.T22N}CG1578^{19} (BDSC 23235); y^1 w*; P{UASp-YFP.Rab5.Q88L}Reph^{24} (BDSC 9774); y^1 w*; P{UASp-YFP.Rab7.Q67L}19 (BDSC 24103); w*; P{UAS-Rab5.S43N}2 (BDSC 42703); y1 w*; P{UASp-YFP.Rab5.S43N}Eip75B02/TM3, Sb1 (BDSC 9772); w[1118]; TI{TI}Rab5[EYFP] (BDSC 62543).

Expression of *UAS-Mv-GFP* and *Rab* alleles was driven by *P{matα4-GAL-VP16}V2H (BDSC 7062)*.

Oregon-R flies were used as a wild-type control.

Cell lines

Human fibroblasts Chediak-Higashi Syndrome (GM02075) and control fibroblasts (AG21862) were obtained from the Coriell Institute for Medical Research and maintained in Eagle's Minimum Essential Medium (Sigma-Aldrich) containing 20% Heat-Inactivated Fetal Bovine Serum (Gibco).

To generate stably transformed cell lines, EGFP-RAB5A.S34N (Addgene #28045) was electroporated into fibroblasts using the Neon Transfection System (Life Technologies) according to the manufacturer's protocol and transformants were selected using G418 antibiotic (Merck).

To generate stable DMEL cell lines expressing inducible Mv-GFP, wild-type cells were transfected with pMT-pCoBlast-Mv-GFP using FuGENE® HD Transfection Reagent (Promega) according to manufaturer's protocols. 48 h after transfections, 25 μg/ml blasticidin was added to the media to select transformed cell lines.

METHODS DETAILS

Cytogenetic mapping of mv alleles

Previous complementation tests had indicated that *Rosario* maps between the proximal breakpoints *Df(3L)Aprt66* and *Df(3L)Aprt198* but at that time there was no gene predicted in this area (Kasravi et al., 1999). We had also found that *fs(3)ros* was allelic with a second site mutation in a gene named *l(3)dre6* (Sliter et al., 1989). Taking advantage of deficiencies *Df(3L)BSC366* and *Df(3L)ED4284* that subsequently became available, we localized *Rosario* to a smaller genomic region between 62B10 and 62B12 (Figure 1B).

Cloning

The BAC (Bacterial Artificial Chromosome) recombineering (recombination-mediated genetic engineering) strategy described in (Venken et al., 2009) was used to insert mCherry or a Tetracysteine Cys-Cys-Pro-Gly-Cys-Cys (TC)- 2xFLAG tags in a frame at the C-terminal end of Mauve in its genomic segment carried in the BAC clone CH322-23O09 (BACPAC Resources Center - BPRC). Briefly, two PCR fragments were generated containing the TC-FLAG or mCherry tags followed by 4 stop codons and a kanamycin resistance gene. Both fragments had 50bp homology arms with Mauve CDS at their N terminus (CAGAGGGGCTCTAT GGAAATGCCCCGAAATTCCCTCAAATCGTCTACAAA) and Mauve 3'UTR at their C terminus (complement of TGTTAATGGGAAA ATACTCAATAATTCAACTCAAAGCATATCAATGACAG) (see Table S1). The kanamycin resistance gene was introduced to facilitate the following colony screening.

The Mv_TC_FLAG_Kana PCR fragment was generated by the following PCR reactions:

1st PCR round: 2XFLAG_4XStop_KanaFF + KanaTagRev

2nd PCR round: 50ntffTC_1xFLAG + KanaTagRev

The Mv_mCherry_Kana PCR fragment was generated by the following PCR reactions:

1st PCR round: 50CDSmCherryFF + KanaStartRev_4Xstop_mCherryRev and mCherryEnd_4XStop_KanaFF+ KanaTagRev

2nd PCR round: 50CDSmCherryFF+ KanaTagRev

The PCR fragment and BAC clone were electroporated into electrocompetent recombineering SW102 cells (NCI Frederick) according to standard protocols and plated on chloramphenicol and kanamycin plates. Single colonies were isolated and BAC clones purified and sequenced to confirm the reading frames. Positive tagged BACs were then transfected into TransforMax™ EPI300™ Electrocompetent *E. coli* cells (Epicentre) for plasmid copy induction, purified using a BACMAX™ DNA Purification Kit (Epicentre) and injected into recipient fly stocks (Fly Facility – Department of Genetics, University of Cambridge).

mauve cDNA was cloned into pDONR221. RNA was extracted from Oregon-R larvae using the RNeasy mini kit (QIAGEN) to provide the template for cDNA synthesis using the SuperScript™ III First-Strand Synthesis System (Thermo Fisher Scientific). The following four *mauve* fragments were amplified from Or-R cDNA and cloned in pDONR221 using the Gateway BP clonase system

(Thermo Fisher Scientific): 1-2230bp, 2161-6241bp, 6121-8221bp, 8101- end. Each fragment was amplified and used for Gibson assembly (NEB) according to manufacturer's protocol. Single colonies were isolated, plasmids were purified and sequenced to confirm the correct full-length cDNA and open reading frames according to the Ensembl database (http://www.ensembl.org/index.html). The *mv* pDONR221 plasmid was then used for the Gateway LR reaction with a modified pPWG vector bearing the *attB* region (pPWG-*attB*) inserted at a *StuI* site to generate a *UAS-Mv-GFP* plasmid that was introduced into *Drosophila* by transformation. To generate the pPWG-*attB* vector, the pPWG vector was digested with *StuI* and treated with Calf Intestinal Alkaline Phosphatase (New England Biolabs) according to the manufacturer's protocol. attBff+attBrev primers were incubated with T4 Polynucleotide Kinase (New England Biolabs) according to the manufacturer's protocol. Linearised pPWG vector and *attB* primers were mixed and ligated using T4 DNA ligase (New England Biolabs) according to the manufacturer's protocol and then transfected into *E. coli ccdB*. Individual colonies were isolated and plasmids purified and sequenced.

The *mv* pDONR221 plasmid was then used for the Gateway LR reaction with a metallothionein-regulated constructs with GFP C-terminal tag (pMT-pCoBlast-Mv-GFP) for expression in DMEL cells.

qPCR

Total RNA was extracted from larvae (*Oregon-R*, *Df(3L)R-G7/TM6B*, mv^{3}*/Df(3L)R-G7* and mv^{ros}*/Df(3L)R-G7*) using the RNeasy mini kit (QIAGEN). Real-time PCR was performed with *Power* SYBR® Green RNA-to-CT™ 1-Step Kit following manufacturer protocol. The *EF1* gene was used as reference gene. Primers are listed in Table S1.

Immunofluorescence and live imaging

0-3h old embryos were dechorionated with 50% bleach and transferred to a 1:1 mix of Methanol:Heptane for devitellinisation and fixation. For immunostaining, embryos were rehydrated in a 1:1 mix of Methanol and 1X PBS for 10 min, washed with PBS + 0,1 % Tween for 15 min, and then blocked with PBS containing 0.1 % Tween and 1% BSA for 30 min and agitated on a spinning wheel at room temperature.

For microtubule regrowth assays, dechorionated embryos were transferred into a tube containing PBS and incubated on ice for 5 min. Upon removal of the PBS, the tube was shifted to room temperature for the indicated amount of time and then embryos were fixed according to the above protocol.

Primary antibody incubation was carried out overnight at 4°C with agitation on a slowly spinning wheel. After 3 washes with PBS containing 0.1 % Tween at room temperature, a secondary antibody was add and incubation continued for 1 h at room temperature in the dark. After 3 washes with PBS containing 0.1 % Tween, embryos were transferred onto a slide with Vectashield Mounting Media with DAPI (Vectorlabs) and sealed under a coverslip.

For immunostaining of ovaries, females were dissected in 0.2% PBT (PBS containing 0.2% Tween) and ovaries were then fixed for 20 min in 4% paraformaldehyde in PBT. After 2 washes in PBT, ovaries were blocked for 1 h with PBT containing 10% BSA before incubation overnight with primary antibody at 4°C in PBS containing 2% Tween. The following day, ovaries were washed 3 times in PBT and then incubated with secondary antibody for 2 h at room temperature. They were then washed 3 times with PBT, transferred onto a slide with Vectashield Mounting Media with DAPI (Vectorlabs) and sealed with a coverslip.

For Nile Red staining, 0-3 h old embryos were were dechorionated with 50% bleach and transferred to a 1:1 mix of 4%Formaldehyde in PBS:Heptane for 20 min. Formaldehyde layer was then removed and replaced with Methanol for 5 min. Embryos were then rehydrated in a 1:1 mix of Methanol and 1X PBS for 10 min, washed with PBS + 0,1 % Tween for 15 min and then blocked with PBS containing 0.1 % Tween and 1% BSA for 30 min before incubation with Nile Red 1μg/ml in PBS for 30 min at room temperature. Embryos were then washed 3 times with PBS (5 min each), transferred onto a slide with Vectashield Mounting Media with DAPI (Vectorlabs), and sealed with a coverslip.

Immunofluorescence images were acquired using a Leica SP8 confocal Microscope with a 40x oil objective.

For live imaging of ovaries and Ub-Tub-RFP Ub- Fzr-GFP embryos, a Zeiss Axiovert 200 microscope equipped with a PerkinElmer RSIII spinning disk confocal unit and running the Volocity v6.3 Software was used. Ovaries were dissected in Voltalef 10S oil and transferred onto a coverslip for imaging. Images were acquired every 2 min (time stamp hh:mm). To image living embryos, 0-1 h old embryos were manually dechorionated and transferred onto a coverslip with glue. Embryos were then covered with Voltalef 10S oil and imaged. Images were acquired every 2 min (time stamp hh:mm) and processed with Huygens Software.

For immunofluorescence of human fibroblasts, cells were grown on coverslips, fixed with cold methanol for 5 min, washed with PBS, blocked for 30 min with PBS containing 1% BSA at room temperature, and then incubated overnight at 4°C with primary antibodies in PBS. After 3 washes with PBS at room temperature, cells were incubated with secondary antibody in PBS for 1 h at room temperature in the dark. After 3 washes with PBS, coverslips were covered in Vectashield Mounting Media containing DAPI (Vectorlabs), mounted onto slides, and imaged using a widefield microscope. Images were processed with Huygens Software.

Antibodies

The following antibodies were used for immunofluorescence: mouse anti α-tubulin (clone DM1A, Sigma-Aldrich) 1:1000; rabbit anti-Msps rb2219 (this study) 1:1000; rabbit anti-Cnn (Bettencourt-Dias et al., 2005) 1:2000; chicken anti-Dplp (Rodrigues-Martins et al., 2007) 1:1000; rabbit anti-mCherry (Abcam ab167453) 1:2000; mouse anti-LAMP1 human (clone H4A3, DSHB) 1:500; anti γ-tubulin (clone GTU-88, Sigma-Aldrich) 1:1000, rabbit anti-Rab5 antibody - *Drosophila* Early Endosome Marker (Abcam ab31261) 1:100; Recombinant rabbit monoclonal Anti-hRab5 antibody (Abcam ab218624) 1:1000

A rabbit anti-Msps was generated against the 1350-1785 amino acid fragment of Msps. DNA encoding the fragment was amplified by PCR from cDNA (Table S1), cloned in the pDONR221 entry vector, and recombined with the pDEST17 destination vector to generate a recombinant DNA encoding a 6xHis N-terminally tagged fusion fragment. The 6xHis fragment was expressed in bacteria, affinity purified on Ni-NTA Agarose beads (Qiagen) and used to for rabbit immunizations (Harlan UK). The final bleed was used for immunostainings and western blots.

Mass spectrometry, Co-IPs, Coomassie staining and Western blots

To purify Mauve-mCherry complexes, 0-3h embryos were collected, dechorionated, and immediately frozen at -80°C. 1 gram of embryos was used for each pulldown experiment. Protein extracts were prepared and isolated using RFP-Trap-MA beads (Chromotek) according to the manufacturer's protocols. Samples were analysed at Laboratory of Mass Spectrometry, IBB PAS (Poland).

For co-immunoprecipitation from embryos, 0-3h old embryos were dounce homogenized in lysis buffer (20 mM Tris-HCl, pH8, 110 mM KCl, 5 mM $MgCl_2$, protease inhibitors) at 4°C and passed through a 30 gauge needle. Lysates were clarified by centrifugation at 20,000 g for 30 min at 4°C. Supernatant was the split in two fractions and added with either 100 μM GDP or GppNHp (Gillingham et al., 2014) and then added to RFP-Trap-MA beads (Chromotek) according to manufacturer's protocol.

For co-immunoprecipitation of Mv-GFP complexes, stable cell lines expressing pMT-pCoBast-Mv-GFP were induced for 24 h with 100μM $CuSO_4$. Wild-type DMEL cells were used as control. Cells were lysated by freeze and thaw and homogenized in lysis buffer (20 mM Tris-HCl, pH8, 110 mM KCl, 5 mM $MgCl_2$, protease inhibitors) at 4°C. Lysates were clarified by centrifugation at 20,000 g for 30 min at 4°C and supernatants were incubated with GFP-Trap-MA beads (Chromotek).

For Western blots, the following antibodies were used: rabbit anti-Msps1:40000, mouse anti α-tubulin (clone DM1A, Sigma Aldrich) 1:10000, rabbit anti p55/dCAF1 (AbCam) 1:2000, rabbit anti-Rab5 antibody - *Drosophila* Early Endosome Marker (Abcam ab31261) 1:5000, rabbit anti-mCherry (Abcam ab167453) 1:1000, rabbit anti-GFP (Abcam ab6556) 1:5000, mouse anti-γ-tubulin (clone GTU-88, Sigma Aldrich) 1:5000

For Coomassie staining of yolk proteins and Western blots, ovaries were isolated from 10 females, boiled in 100μl of 2X SDS Loading Buffer. 10μl were loaded for SDS-polyacrylamide gel electrophoresis and processed according to standard protocols.

For Coomassie of yolk uptake in embryos, embryos were collected, protein extracts were prepared and quantified. 50μg of total protein extracts were loaded for each genotype.

Illustrations and figure preparation

Graphical illustrations were done with Adobe Illustrator Software and figures were assembled using Adobe Photoshop Software.

QUANTITATIONS AND STATISTICAL ANALYSIS

To measure yolk granule diameters, embryos were dechorionated using 50% bleach, transferred onto a coverslip, covered with glycerol and immediately imaged using a Leica SP8 Confocal Microscope with a 40X oil objective and the 405 nm wavelength laser. Individual diameters were then manually measured using Fiji/ImageJ software (Schindelin et al., 2012), on at least 5 different embryos for each genotype; a minimum of 20 representative yolk granules were measured in each embryo.

To measure Lamp1 diameter in fibroblasts, pictures of immunostained fibroblasts were manually measured using Fiji/ImageJ software and measurements were analyzed with GraphPad software.

Fluorescence intensity measurements were made from images using Fiji/ImageJ. A circle of a fixed diameter was used to measure fluorescence intensity at centrosomes and an area without centrosomes within the same embryo was used to determine background to be subtracted from the measurements. At least 5 different embryos for each genotype were quantified and at least 20 representative centrosomes were scored from each embryo. Measurements were then transferred to GraphPad software for statistical analysis.

Quantification of YPs from Coomassie staining was carried out using Fiji/ImageJ software. For each lane, the intensity of YP band was measured (top yellow box in Figure 2B) together with intensity with a reference band (ref, bottom yellow box in Figure 2B). Arbitrary units (AU) represent the ratio between intensity of YP band and the reference band.

Statistical parameters of individual experiments (value of n, mean, SEM, p value) are reported in each figure legend in the paper.

Developmental Cell, Volume *56*

Supplemental information

Mauve/LYST limits fusion of lysosome-related organelles and promotes centrosomal recruitment of microtubule nucleating proteins

Ramona Lattao, Hélène Rangone, Salud Llamazares, and David M. Glover

Figure S1

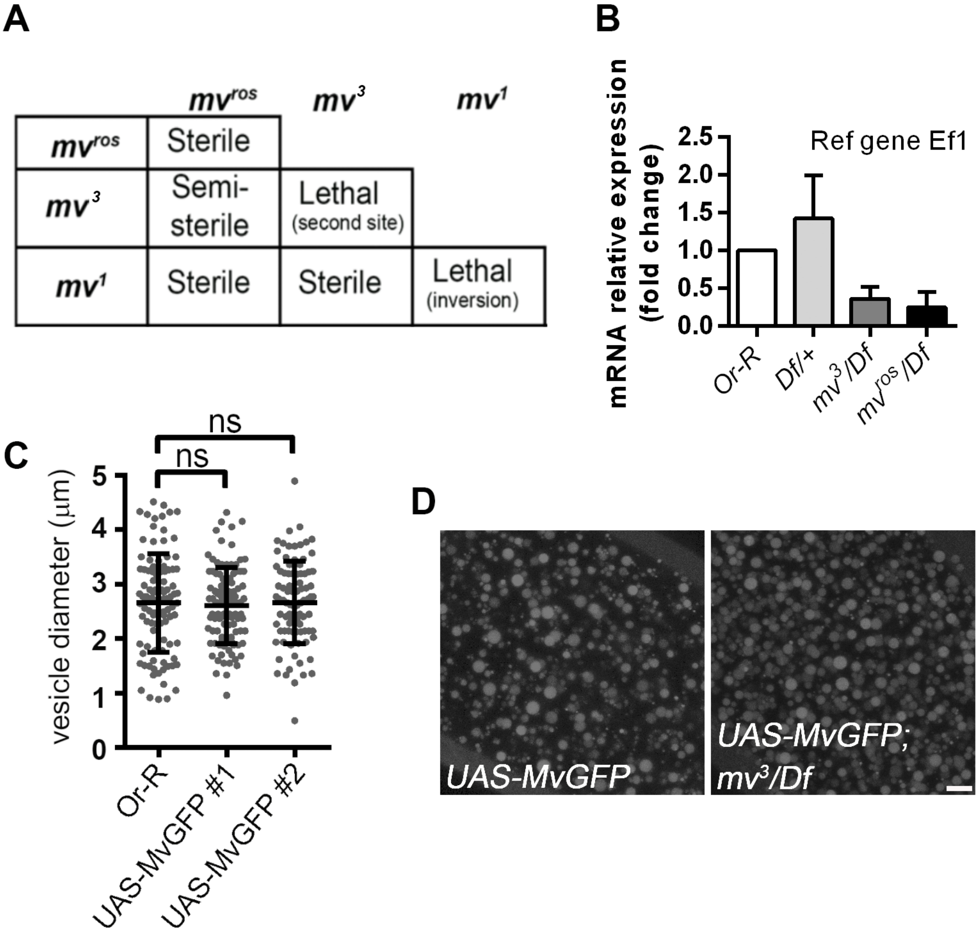
A
mv^ros
mv^3
mv^1
mv^ros
Sterile
mv^3
Semi-sterile
Lethal (second site)
mv^1
Sterile
Sterile
Lethal (inversion)
B
mRNA relative expression (fold change)
Ref gene Ef1
2.5
2.0
1.5
1.0
0.5
0.0
Or-R
Df/+
mv3/Df
mvros/Df
C
ns
ns
vesicle diameter (μm)
5
4
3
2
1
0
Or-R
UAS-MvGFP #1
UAS-MvGFP #2
D
UAS-MvGFP
UAS-MvGFP; mv3/Df

Figure S1 related to Figure 1. Identification of *mauve* alleles

(A) Phenotypes of different allelic combinations of *mauve* (*mv*)

(B) Quantitation of mauve mRNA level via qPCR in *Or-R*, *Df/+*, mv^{3}*/Df* and mv^{ros}*/Df*. The EF1 gene was used as reference.

(C) Vesicle diameter of YGs from *Or-R* embryos and 2 *UAS-MvGFP* lines (#1 and #2) driven by the maternal tubulin GAL4 promoter *P{matα4-GAL-VP16}* show no significative differences. Unpaired t test : *Or-R UAS-MvGFP #1* P value = 0,6425. *Or-R UAS-MvGFP #2* P value = 0,9430 (n=100, N=5)

(D) Examples of YGs from embryos of the indicated genotypes driven by *P{matα4-GAL-VP16}*. Scale bar =10 µm

Figure S2

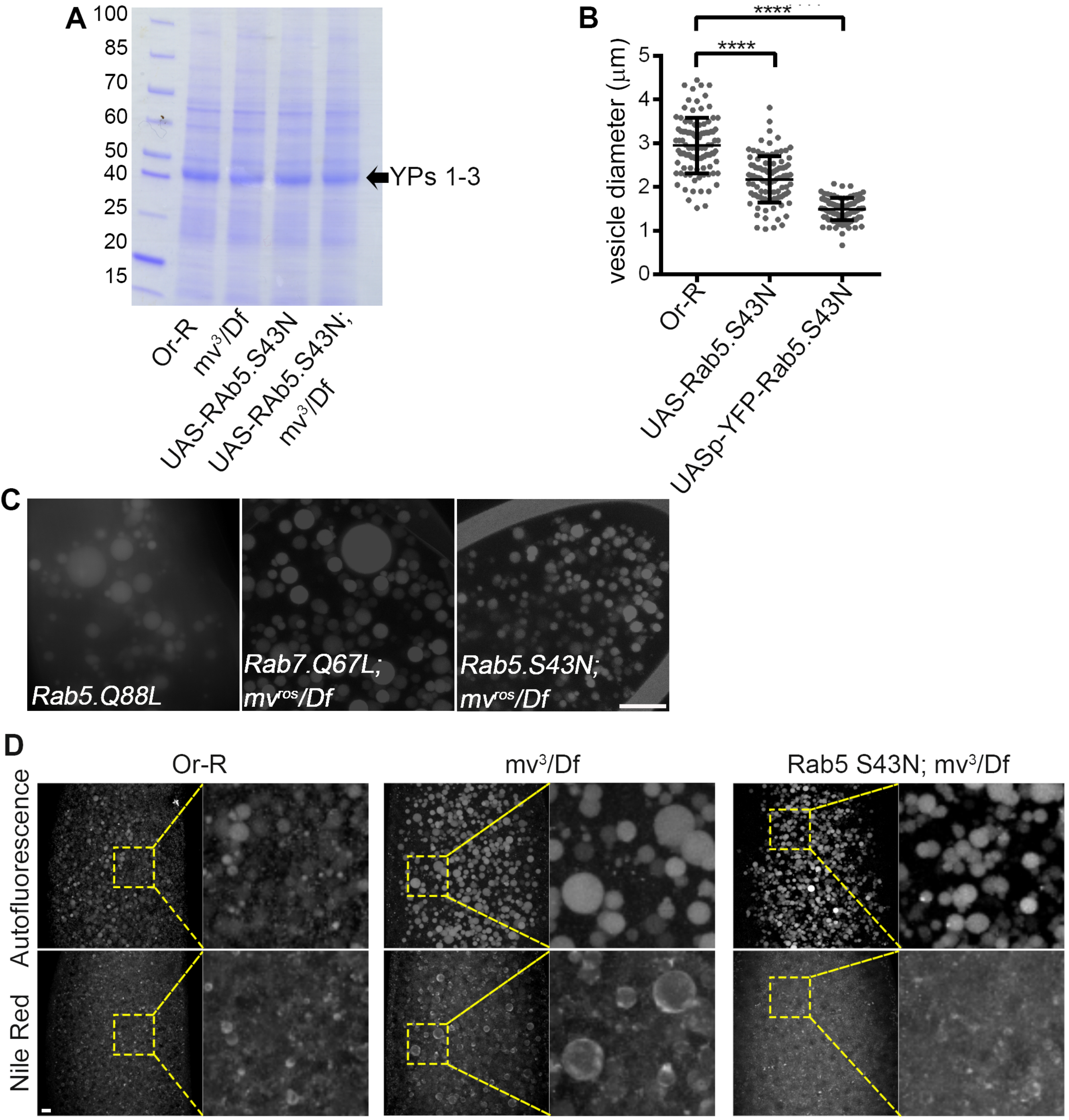
A
100
85
70
60
50
40
25
20
15
YPs 1-3
Or-R
mv3/Df
UAS-RAb5.S43N
UAS-RAb5.S43N;
mv3/Df
B
****
****
vesicle diameter (μm)
0
1
2
3
4
5
Or-R
UAS-Rab5.S43N
UASp-YFP-Rab5.S43N
C
Rab5.Q88L
Rab7.Q67L;
mvros/Df
Rab5.S43N;
mvros/Df
D
Or-R
mv3/Df
Rab5 S43N; mv3/Df
Autofluorescence
Nile Red

Figure S2 related to Figure 2. Rab5.S43N does not affect yolk uptake and reduces fusion between YGs and LDs

(A) Coomassie staining of embryos of the indicated genotypes shows no defects in yolk uptake. 50µg of total embryonic extracts were loaded in each well. YPs band is the strongest band between 40 and 50 KDa. Expression of UAS transgenic constructs was driven by the maternal tubulin GAL4 promoter *P{matα4-GAL-VP16}*

(B) Vesicle diameter of YGs from embryos of the indicated genotypes to compare effect of 2 different Rab5.S43N transgenes with two different UAS promoters. Expression of UAS transgenic constructs was driven by *P{matα4-GAL-VP16}*. n=100, N=5 Unpaired t test: **** = P<0.0001. In all of our experiments, we used the UAS-Rab5.S43N transgene that has milder expression compare to the UASp driven transgene

(C) Examples of YGs from embryos of the indicated genotypes driven by *P{matα4-GAL-VP16}*. Scale bar =20 µm. See also Figure 2F, G

(D) Related to Figure 2H. Nile red staining of lipid droplets (LDs) and autofluorescent YGs in *Or-R*, *mv*^{3}*/Df* and *matα4-GAL-VP16/UAS-Rab5.S43N; mv*^{3}*/Df* embryos. In wild-type embryos, Nile Red show small puncta and sporadic localization around autofluorescent YG. In *mv*^{3}*/Df* there is a significative increase in Nile Red staining around YG that decreases in *matα4-GAL-VP16/UAS-Rab5.S43N; mv*^{3}*/Df* embryos. (n=50) Scale bar =10 µm

Figure S3

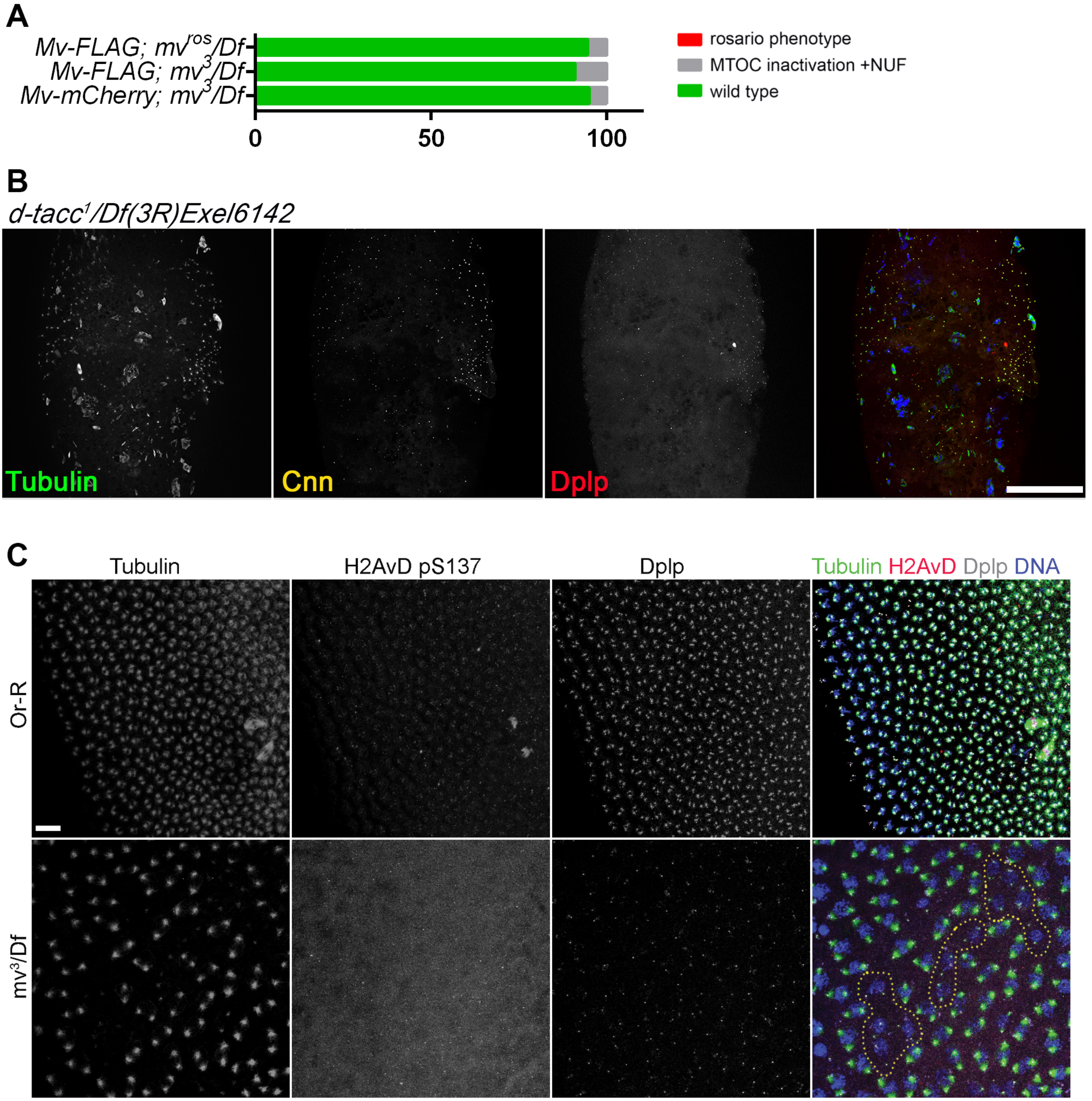
A
Mv-FLAG; mvros/Df
Mv-FLAG; mv3/Df
Mv-mCherry; mv3/Df
0
50
100
rosario phenotype
MTOC inactivation +NUF
wild type
B
d-tacc1/Df(3R)Exel6142
Tubulin
Cnn
Dplp
C
Tubulin
H2AvD pS137
Dplp
Tubulin H2AvD Dplp DNA
Or-R
mv3/Df

Figure S3 related to Figure 3. *d-tacc* mutants show a rosario-like phenotype and MTOC inactivation and NUF in mauve mutants are not consequences of DNA damage

(A) Frequencies (mean value) of different classes of phenotype after rescue with two different Mv transgenes (*Mv-mCherry* and *Mv-FLAG.*) n>100 for each genotype. See also Figure 3B

(B) Embryo from *d-tacc^{1}/Df(3R)Exel6142* female showing a rosario-like phenotype. Staining reveals α-tubulin, green; Centrosomin (Cnn), white; Dplp, red; and DNA, blue. Scale bar= 50µm

(C) *Or-R* and *mv^{3}/Df* embryos stained for α-tubulin (green), H2AvD pS137 (red), Dplp (grey) and DAPI (blue) show that MTOC inactivation in *mauve* mutants (yellow dotted area) is not a consequence of DNA damage. Scale bar = 10µm

Figure S4

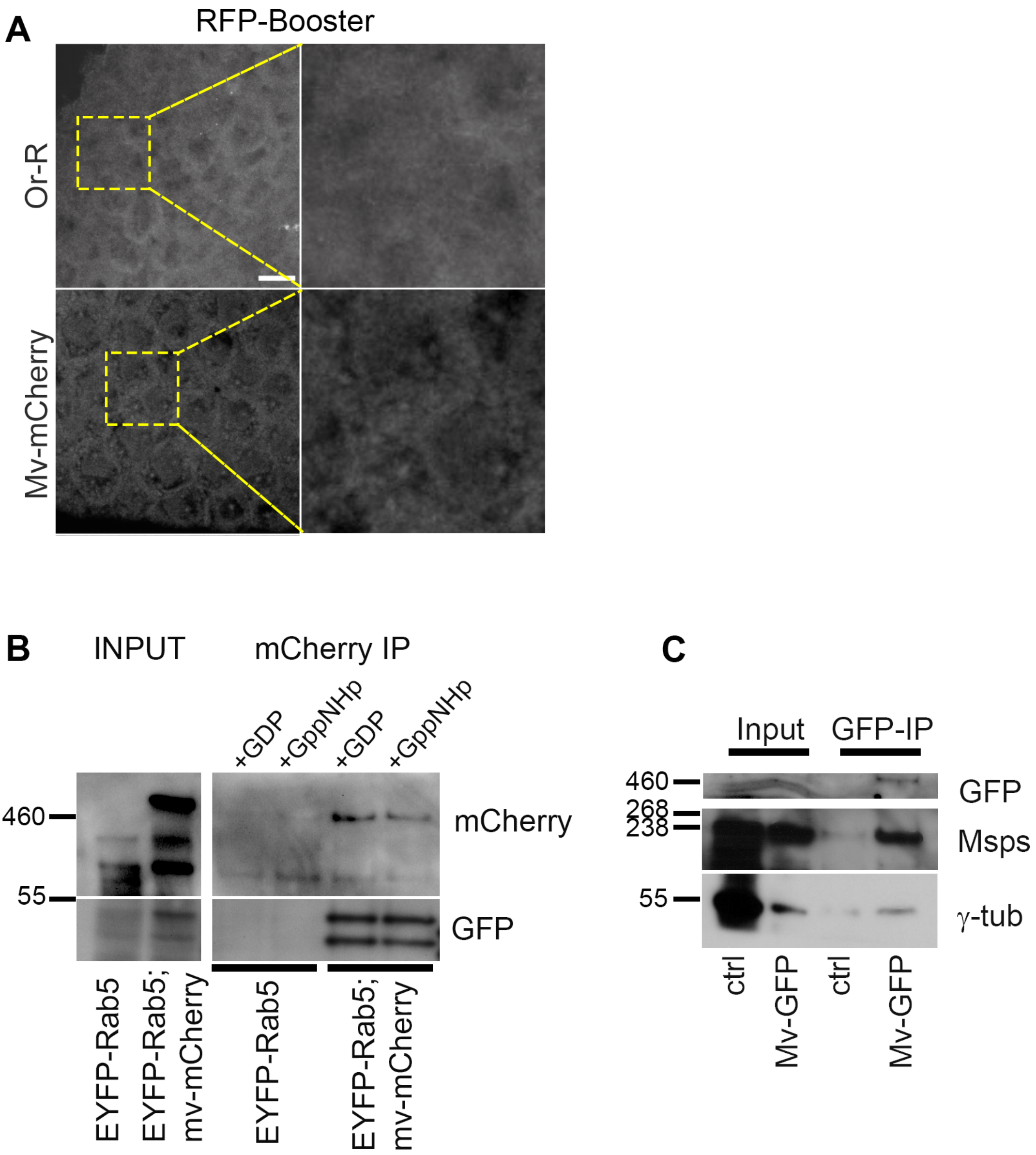
A
RFP-Booster
Or-R
Mv-mCherry
B
INPUT
mCherry IP
+GDP
+GppNHp
+GDP
+GppNHp
460
55
mCherry
GFP
EYFP-Rab5
EYFP-Rab5; mv-mCherry
EYFP-Rab5
EYFP-Rab5; mv-mCherry
C
Input
GFP-IP
460
268
238
55
GFP
Msps
γ-tub
ctrl
Mv-GFP
ctrl
Mv-GFP

Figure S4 related to Figure 4C, D. Mauve localizes at the mitotic spindle and interacts with Rab5, Msps and γ-tubulin

(A) Related to Figure 4C. Mv-mCherry localization in early embryonic divisions revealed with RFP-Booster Alexa Fluor® 568 (Chromotek). Mv-mCherry localizes all over the mitotic spindles and it enriches at spindle poles. Scale bar = 50µm

(B) Co-immunoprecipitation of Mv-mCherry complexes from 0-3h old embryos co-expressing Mv-Mcherry and EYFP- Rab5 (embryos expressing EYFP-Rab5 alone were used as control) in presence of GDP or the non-hydrolyzable GTP analog GppNHp. Importantly, EYFP-Rab5 is expressed under the endogenous promoter (*w[1118]; TI{TI}Rab5[EYFP]*, BDSC 62543) to avoid overexpression. Mv interacts *in vivo* with Rab5 and the interaction is not affected by the GDP/GTP conformation of Rab5.

(C) Co-immunoprecipitation of Mv-GFP complexes from DMEL cells in absence of detergent. wild-type DMEL cells were used as control. DMEL cells stable transfected with Mv-GFP under a metallothionein promoter were induced for 24h with 100µM $CuSO_4$ before Co-IP. Endogenous Msps and γ-tubulin were detected in the Mv-GFP IP but not in the control IP.

Figure S5

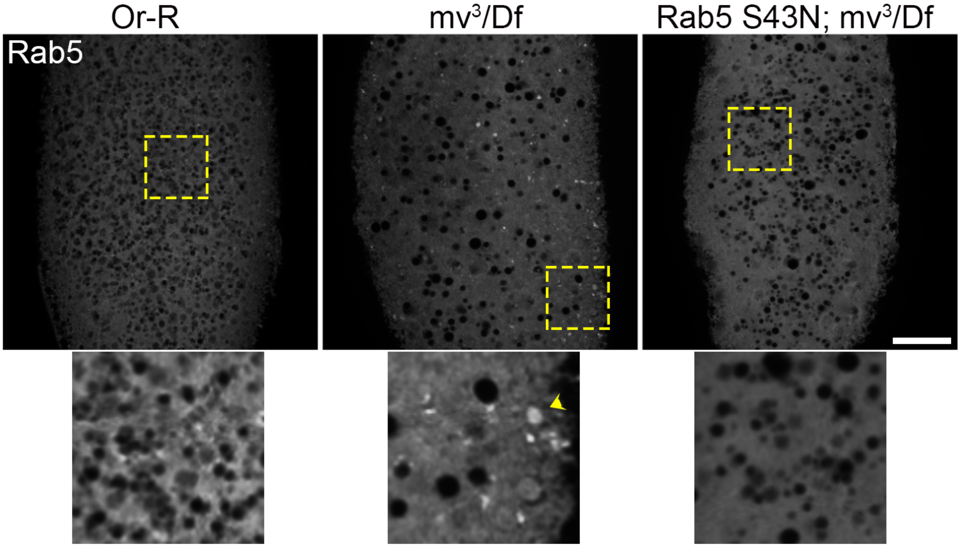

Figure S5 related to Figure 5 Rab5 accumulation around YGs in *Drosophila* embryos

In wild-type embryos, small puncta of Rab5 are interspersed within the YGs. In *mauve* mutant embryos, bigger Rab5 positive bodies accumulates in the vicinity of but are not incorporated into the YG “ghosts”. The accumulation of such bodies was suppressed by Rab5.S43N. Scale bar = 50 µm, insert = 3X

Figure S6

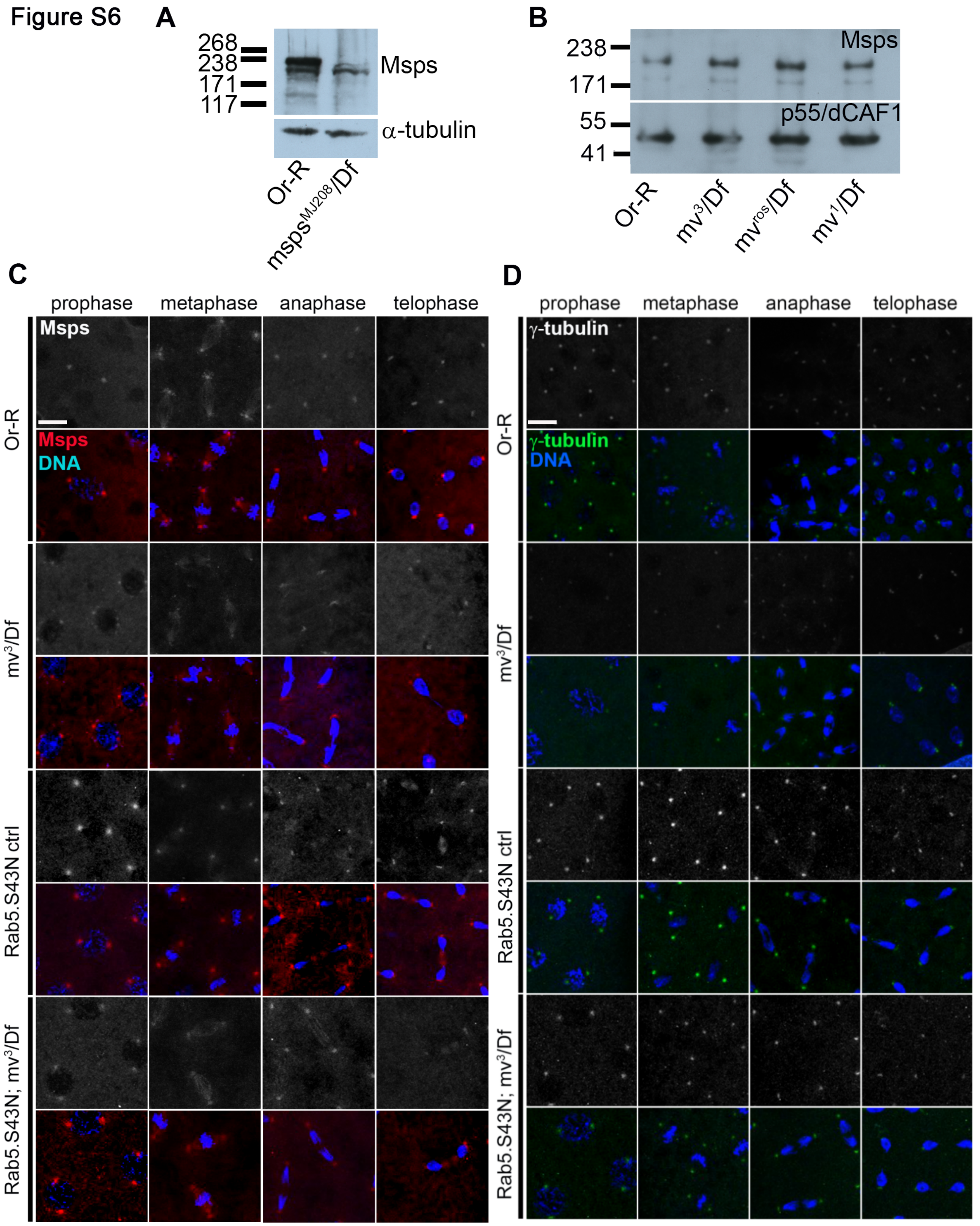
A
268
238
171
117
Msps
α-tubulin
Or-R
msps^MJ208/Df
B
238
171
55
41
Msps
p55/dCAF1
Or-R
mv3/Df
mvros/Df
mv1/Df
C
prophase
metaphase
anaphase
telophase
Msps
Msps
DNA
Or-R
mv3/Df
Rab5.S43N ctrl
Rab5.S43N; mv3/Df
D
prophase
metaphase
anaphase
telophase
γ-tubulin
γ-tubulin
DNA
Or-R
mv3/Df
Rab5.S43N ctrl
Rab5.S43N; mv3/Df

Figure S6 related to Figure 6. Characterization and staining with anti-Msps antibody

(A) Validation of Rabbit anti-Msps antibody raised against the 1350-1785 amino acid fragment of Msps. Western blots of ovary extracts from *Or-R* and $msps^{MJ208}$/*Df* flies. $msps^{MJ208}$ is a hypomorphic allele of Msps. Note the significative reduction in the band corresponding to Msps. α-tubulin was used as loading control.

(B) Western blots of ovary extracts of *Or-R*, mv^{3}/*Df*, mv^{ros}/*Df* and mv^{1}/*Df* flies show that total levels of Msps are not affected in mauve mutants. p55/dCAF1 was used as loading control.

(C) Related to Figure 6C and showing all stages of mitosis stained to reveal Msps and DNA. Scale bar= 10µm

(D) Related to Figure 6D. Embryos from mothers of the indicated genotypes at representative stages of mitosis stained to reveal γ-tubulin and DNA. Scale bar= 10µm

Figure S7

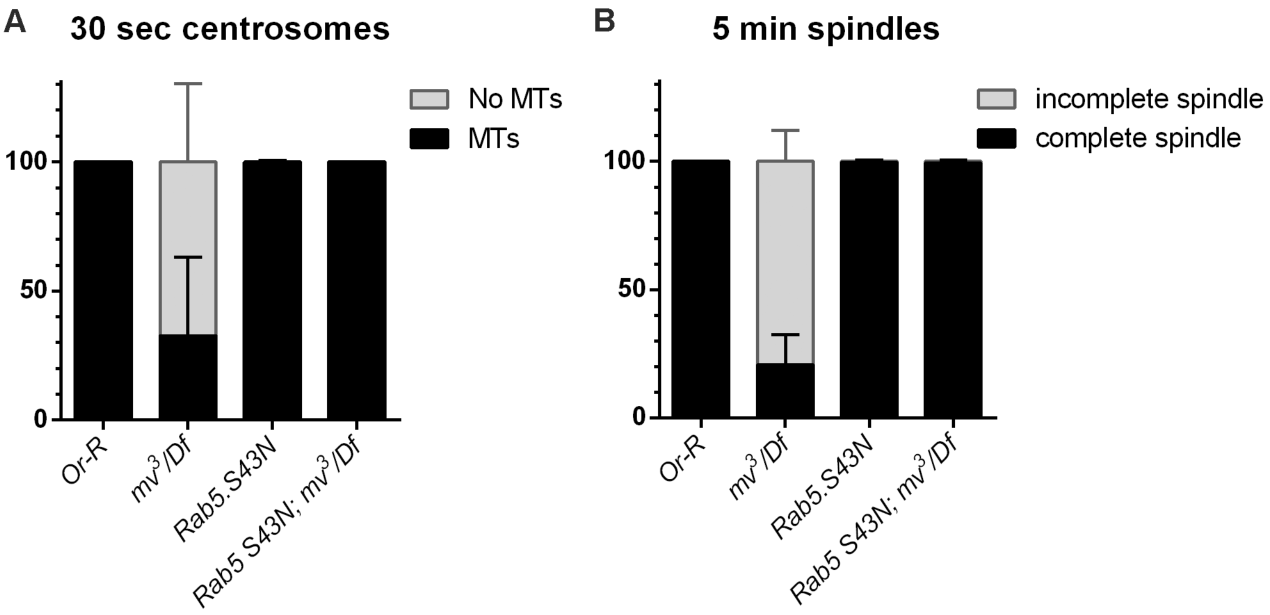
A
30 sec centrosomes
No MTs
MTs
100
50
0
Or-R
mv3/Df
Rab5.S43N
Rab5 S43N; mv3/Df
B
5 min spindles
incomplete spindle
complete spindle
100
50
0
Or-R
mv3/Df
Rab5.S43N
Rab5 S43N; mv3/Df

Figure S7 related to Figure 7. Quantification of MTs regrowth at 2 time points: 30 sec regrowth and 5 min regrowth

(A) Percentage of spindles with centrosome nucleating/non nucleating MTs after 30 sec regrowth. (*Or-R* n=124, N=4; *mv*^{3}*/Df* n=86, N=4; *Rab5.S43N* n=141, N=4; *Rab5.S43N mv*^{3}*/Df* n=184, N=5)

(B) Percentage of spindle with a full regrown spindle (complete spindle) or incomplete spindle after 5 min regrowth. (*Or-R* n=171, N=4; *mv*^{3}*/Df* n=160, N=4; *Rab5.S43N* n=126, N=3; *Rab5.S43N mv*^{3}*/Df* n=260, N=5)

Supplemental Table S1 related to STAR Methods. Primers used in this study

PRIMER NAME	SEQUENCE	SOURCE
2XFLAG_4XStop_KanaFF	GACTACAAGGACGATGCGACAAGGACTACAAGGACGATGACGACAAGTAATAATAGTGAGAGTCGTATTACATGGTCATAGC	Sigma-Aldrich
50ntffTC_1xFLAG	CAGAGGGGCTCTATGGAAATGCCCCGAAATTCCCTCAAATCGTCTACAAATGCTGCCCCGGCTGCTGCGACTACAAGGACGATGACGACAAG	Sigma-Aldrich
KanaTagRev	CTGTCATTGATATGCTTTGAGTTGAATTATTGAGTATTTTCCCATTAACATTAGTCCCGTCAAGTCAGCGTATT	Sigma-Aldrich
50CDSmCherryFF	CAGAGGGGCTCTATGGAAATGCCCCGAAATTCCCTCAAATCGTCTACAAAATGGTGAGCAAGGGCGAGG	Sigma-Aldrich
KanaStartRev_4Xstop_mCherryRev	GCTATGACCATGTAATACGACTCTCACTATTATTACTTGTACAGCTCGTCCATGCC	Sigma-Aldrich
mCherryEnd_4XStop_KanaFF	GCATGGACGAGCTGTACAAGTAATAATAGTGAGAGTCGTATTACATGGTCATAGC	Sigma-Aldrich
attBff	CGGCGGTGCGGGTGCCAGGGCGTGCCCTTGGGCTCCCCGGGCGCGTACTCCAC	Sigma-Aldrich
attBrev	GTGGAGTACGCGCCCGGGGGCCCAAGGGCACGCCCTGGCACCCGCACCGCGG	Sigma-Aldrich
qPCR_Mv_ff	GGATATCAAGCTCATTGC	Sigma-Aldrich
qPCR_Mv_rev	GTCCCAGAGCAAAGTTATG	Sigma-Aldrich
qPCR_EF1_ff	GCGTGGGTTTGTGATCAGTT	Sigma-Aldrich
qPCR_EF1_rev	GATCTTCTCCTTGCCCATCC	Sigma-Aldrich
Msps_1350-1785_Nter_ff	GGGGACAAGTTTGTACAAAAAAGCAGGCTTC TCG GCG GTG CGC GAG ATT GCT C	Sigma-Aldrich
Msps_1350-1785_Nter_rev	GGGGACCACTTTGTACAAGAAAGCTGGGTCcta CTC GAG AAT TAC GGC GTC ATA GTT C	Sigma-Aldrich
